# County-Level Variation in Changes in Firearm Mortality Rates Across the US, 1989 to 1993 vs 2015 to 2019

**DOI:** 10.1001/jamanetworkopen.2022.15557

**Published:** 2022-06-06

**Authors:** Michelle Degli Esposti, Jason Gravel, Elinore J. Kaufman, M. Kit Delgado, Therese S. Richmond, Douglas J. Wiebe

**Affiliations:** 1 Penn Injury Science Center, University of Pennsylvania, Philadelphia; 2 Department of Social Policy and Intervention, University of Oxford, Oxford, United Kingdom; 3 Department of Criminal Justice, Temple University, Philadelphia, Pennsylvania; 4 Division of Traumatology, Surgical Critical Care, and Emergency Surgery, University of Pennsylvania Perelman School of Medicine, Philadelphia; 5 Department of Biostatistics and Epidemiology, University of Pennsylvania Perelman School of Medicine, Philadelphia; 6 Department of Emergency Medicine, University of Pennsylvania Perelman School of Medicine, Philadelphia; 7 Biobehavioral Health Sciences Department, University of Pennsylvania School of Nursing, Philadelphia

## Abstract

**Importance:**

Firearm violence remains a critical public health challenge, disproportionately impacting some US regions. County-level variation may hold key insights into how firearm mortality rates vary across the US.

**Objective:**

To model county-level changes in firearm mortality rates (total, homicide, and suicide) from 1989 to 1993 vs 2015 to 2019 and identify and characterize hot spots showing unexpected changes over time.

**Design, Setting, and Participants:**

This is a cross-sectional study with 2 time points using a novel small area estimation method to analyze restricted access mortality microdata by cause of death and US county. The analysis included 3111 US counties from 49 states and the District of Columbia from January 1, 1989, to December 31, 2019. Bayesian spatial models were fitted to map geographical variation in changes in age-standardized firearm mortality rates (per 100 000 person-years) from 1989 to 1993 vs 2015 to 2019. County outliers (or hot spots) were defined as having observed rates that fell outside the 95% credible intervals of their expected posterior predictive distribution. These counties were characterized using visualization and descriptive statistics of their characteristics. Data were analyzed from June to December 2021.

**Exposures:**

County of residence.

**Main Outcomes and Measures:**

Five-year age-standardized mortality rates by US county, age, and cause of death for 1989 to 1993 and 2015 to 2019.

**Results:**

Between 1989 and 2019, 1 036 518 firearm deaths were recorded in counties across the US. Suicide was the most common cause of firearm mortality (589 285 deaths) followed by homicide (412 231 deaths). Age-standardized rates (deaths per 100 000 individuals) for firearm deaths and suicides increased from 1989 to 1993 vs 2015 to 2019 (mean [SD] change, 0.16 [8.78] for firearm deaths and 1.21 [6.91] for suicides), while firearm homicides decreased (mean [SD] change, −0.39 [3.96]). However, these national trends were not homogeneous across counties and often varied by geographical region. The West and Midwest showed the most pronounced increases in firearm suicide rates, whereas the Southeast showed localized increases in firearm homicide rates, despite the national decreasing trend. Critical hot spots were identified in urban counties of Alabama, and firearm homicide rates (per 100 000) in Baltimore City, Maryland, almost doubled from 29.71 to 47.43, and by 2015 to 2019 it accounted for 66.7% of all firearm homicide in Maryland. By contrast, District of Columbia showed promising improvements over time, decreasing from 56.5 firearm homicides per 100 000 in 1989 to 1993 to 14.45 in 2015 to 2019.

**Conclusions and Relevance:**

There was substantial variation in rates and changes in firearm deaths among US counties. Geographical hot spots may be useful to inform targeted prevention efforts and local policy responses.

## Key Points

**Question:**

What are the geographical patterns and hot spots for changes in firearm deaths across the US from 1989 to 2019?

**Findings:**

This cross-sectional study of 1 036 518 firearm deaths among US counties identified pronounced increases in firearm suicide in the West and Midwest and localized increases in firearm homicide in the Southeast. These localized increases, or county hot spots, accounted for a substantial burden of firearm homicide nationally.

**Meaning:**

Analysis of county-level variation in firearm mortality rates offers new insights on how the problem of firearm violence differs across the US.

## Teaser

This cross-sectional study evaluates changes in US firearm mortality rates at the county level, comparing data from 1989 to 1993 vs 2015 to 2019.

## Introduction

In the US, 39 707 people lost their lives to firearms in 2019, including 23 941 suicides and 14 414 homicides.^1,2^ Despite decreases in the 1990s and early 2000s, death rates from firearm injuries have increased in recent years, and 2019 was the third consecutive year in which deaths approached 40 000.^3^ Firearms account for approximately 50% of suicides and 75% of homicides, making firearm injuries the leading cause of violent death and costing the US more than $1 billion a year on hospital costs alone.^4,5^ Yet, there has been limited progress in reducing these preventable deaths and addressing this critical public health problem.^6,7,8^

Although firearm violence is a national problem, firearm injuries and deaths vary greatly by state,^9^ urbanicity,^10,11^ and from city to city.^12,13^ Most US-wide studies have been conducted at the state level, which may be obscuring important geographical variation in rates and changes in firearm deaths over time.^9,10^ So far, limited data availability and sparse data problems (ie, zero and low counts) have prevented detailed spatial analyses of all US counties,^14^ with only a few recent studies using methods that overcome these analytical obstacles.^15^ A more detailed understanding of localities that experience a disproportionate burden of firearm deaths (ie, geographical hot spots) is thus needed to inform service provision and effectively respond to changes in firearm violence in the US.

Importantly, the reasons for population-level differences in firearm deaths are not fully known. Previous studies have adopted a deductive approach to assess hypothesized causes of firearm deaths and explain the geographical and temporal variation in firearm mortality rates.^10,16,17^ Stronger firearm policy environments have been associated with lower rates of firearm deaths,^18,19^ as have specific laws (eg, regulation of firearm dealers^20,21^ and background checks for handgun sales^22,23^) and state characteristics (eg, urbanicity,^10^ income inequality,^24^ and federal firearms licenses^25^). In practice, however, states tend to have several types of laws in place at once, making it difficult to determine the impact of any single law.^26,27^ Moreover, state-level analyses fail to identify within-state variability and cannot account for the impact from local initiatives, changes in built environment, economic trends, population characteristics, culture, and enforcement.^28,29^ An alternative inductive approach is needed to identify smaller geographical units, such as counties, that show unexpected changes in firearm mortality rates and to explore whether these counties are characterized by differences in population sociodemographics, geography, economics, policy, or programming.^30^ Investigating county-level variation may therefore shed new insights on firearm violence which would be lost by relying on deductive approaches alone.

We use an inductive, data-driven, hot-spotting approach as an alternative for understanding county-level variation in firearm violence across the US.^30^ We apply a novel small-area method^31,32^ to identify and characterize counties that showed unexpected changes in firearm mortality rates (total, homicide, and suicide) over time. By learning from these counties, we aim to gain new knowledge of factors leading to local upsurges and reductions in firearm deaths, and thus guide future opportunities for intervention.

## Methods

This descriptive cross-sectional analysis used firearm mortality data from 3111 counties and 49 states and the District of Columbia from January 1, 1989, to December 31, 2019. We followed recommendations set out in the Strengthening the Reporting of Observational Studies in Epidemiology (STROBE) reporting guideline. The research received institutional review board approval from the University of Pennsylvania and was preregistered at the Open Science Framework. Because the data source is publicly available and the data are anonymous, the study was exempt from the need for informed consent in accordance with 45 CFR §46.102(f). Data were analyzed from June to December 2021.

### Data

We analyzed restricted access mortality data from the National Vital Statistics System.^33^ These data are based on deidentified death records of underlying cause of death for more than 99% of all deaths in the US, according to the *International Classification of Diseases, Ninth Revision (ICD-9)* and *International Statistical Classification of Diseases and Related Health Problems, Tenth Revision (ICD-10)*. Information on time of death, county of residence at time of death as a proxy for county of death,^34,35^ and age group (0-19, 20-34, 35-54, and ≥55 years) was included for each decedent, as well as corresponding population estimates. We created a panel data set of space-time observations, with each observation representing 1 year of data per county for a total of 96 441 county-years (3111 counties × 31 years), which was then condensed into a panel data set with two 5-year intervals at the beginning (1989-1993) and end (2015-2019) of the study period. The resulting aggregated data set consisted of 2 temporal observations per county (3111 counties × two 5-year periods); see the “Firearm Mortality Rates” section below for details.

Alaska, Bedford City (Virginia), and Broomfield County (Colorado) were excluded because of definitional inconsistencies during the study period.^36^ Although the counties of contiguous US states remained nearly unchanged, the geographical subdivision of Alaska changed frequently, meaning that county comparisons within Alaska would not be comparing the same geographical regions or populations. For example, in 2000 alone, Alaska deleted 3 counties, introduced 6 new counties, and made 1 substantial boundary change.^37^ Death records that had missing information on the decedent’s age were excluded (1033 decedents), resulting in a complete case analysis of more than 99.9% of all death records.^38^

### Firearm Mortality Rates

We condensed the panel data set to two 5-year intervals at the study period beginning (January 1, 1989, to December 31, 1993) and end (January 1, 2015, to December 31, 2019). Five-year periods were used to minimize 0 and low counts while examining change over a 27-year period. Summed totals were used to derive county-level rates, including age-standardized rates using the direct method and the 2000 population standard^39^ and age-specific rates for each age group. We calculated rates for total firearm death (homicide, suicide, and unintentional and undetermined intent), as well as separately for the 2 main causes of homicide and suicide (see eTable 1 in the Supplement for the *ICD-9* and *ICD-10* codes). We did not separately analyze unintentional deaths or undetermined intent since these accounted for fewer than 3% and 1% of all firearm deaths, respectively.

We estimated expected age-standardized firearm mortality rates in 2015 to 2019 for each county using previous age-standardized rates in 1989 to 1993 for each county. Our modeling approach thus used county-level information on firearm mortality rates in 1989 to 1993 to estimate more recent firearm mortality rates in 2015 to 2019. If counties followed typical expected change, then previous rates would be good indicators of recent rates; if counties showed unexpected change over time, then previous rates would be poor indicators (eFigure 1 in the Supplement). Thus, the deviation between the observed and expected mortality rates in 2015 to 2019 captures unexpected change from 1989 to 1993 vs 2015 to 2019. We used 1989 to 1993 as our baseline period because of limited availability of data for prior years and the advantages of maximizing the window of time between the baseline and current period. Substantial changes to firearm polices occurred early in the first 10 years of this 27-year window of change, accounting for 63% of approximately 172 firearm policy implementations (main policy changes: 36 background checks, 2 carrying a concealed weapon, 46 Castle Doctrine, 6 child access, 54 minimum age, 6 open carry, 6 firearm registration, and 8 sales restriction laws).^40^ The potential to examine the outcomes of these firearm policies would be missed if we restricted the study period.

### County-Level Characteristics

County-level information on geography (rural-urban continuum and land area), sociodemographics (age, sex, race, and ethnicity distribution), education (percentage of high school graduates), economics (household income, unemployment rate, and percentage living in poverty), politics (percentage Republican voters), health (percentage heavy drinkers and access to trauma centers), and federal firearm licensed dealers (federal firearm licenses) were collected from diverse federal agencies. Because we were interested in explaining change in firearm mortality rates from 1989 to 1993 vs 2015 to 2019, we collected covariates that measured county characteristics during the period of change (ie, 1994-2014), with most measured in 2005. More details on each measure and their timings are provided in a schematic directed acyclical graph in eFigure 2 and eTable 2 in the Supplement.

### Statistical Analysis

Bayesian spatially explicit regression models were fitted separately for all outcomes (firearm deaths, firearm homicide, and firearm suicide). Bayesian modeling is a flexible and robust approach that can account for sparse data (eg, 0 and low counts) by borrowing strength from neighboring data-rich geographical areas.^41^ The models can also explicitly account for spatial autocorrelation via the Besag-York-Mollié model.^42,43^ We modeled a spatially structured random effect that smoothed the data according to an adjacency matrix for neighboring counties if they share at least 1 common boundary (ie, queen adjacency).^42,43^ Technical details are specified in eMethods in the Supplement. Models were fitted using the Integrated Nested Laplace Approximation method in R statistical software version 4.1.0 (R Project for Statistical Computing), a novel and computationally efficient approach for performing approximate bayesian inference.^44,45^

We compared observed firearm mortality rates 2015 to 2019 with expected rates estimated by our bayesian models. Discrepancies between observed and expected rates indicated whether a county showed lower or higher than expected changes in firearm mortality rates from 1989 to 1993 vs 2015 to 2019. We compared observed rates with the posterior predictive distribution of expected rates for each county and identified counties that had observed firearm mortality rates that fell outside their estimated (2-sided) 95% credible intervals (CrI).^46,47^ Low outliers were defined as counties that had observed values smaller than their lower bound (2.5% CrI), whereas high outliers were defined as counties that had observed values larger than their upper bound (97.5% CrI). Low county outliers represented unexpected improvements, whereas high county outliers represented unexpected deteriorations in firearm mortality rates over time. We described the characteristics of each low and high county outlier and investigated differences between counties that were not identified to be outliers and low and high outliers. We used the Kruskal-Wallis rank sum test to investigate differences in county characteristics by outlier status (no outlier, low outlier, or high outlier), which we supplemented with post hoc pairwise comparisons for characteristics that were identified to be meaningful.^48^ Tests were 2-sided and significance was set at P <. 05. Throughout, we adjusted for multiple testing using the Benjamini-Hochberg method to control for the false discovery rate.^49^

## Results

There were 1 036 518 firearm deaths in 3111 US counties from January 1, 1989, through 31 December, 2019 (eTable 3 in the Supplement). Suicide was the most common cause of firearm mortality, accounting for 589 285 deaths (56.9%) whereas homicide accounted for 412 231 firearm deaths (39.8%). Only a small proportion of firearm deaths were unintentional (25 428 deaths [2.5%]) and even fewer were of undetermined intent (9574 deaths [0.9%]). Age-standardized rates for all firearm deaths increased from 13.97 deaths per 100 000 people in 1989 to 1993 to 14.13 deaths per 100 000 people in 2015 to 2019 (mean [SD] change, 0.16 [8.78]). This was driven by overall increases in firearm suicide rates (mean [SD] change, 1.21 [6.91]) as firearm homicide rates decreased during this period (mean [SD] change, −0.39 [3.96]). Firearm homicide decreased over time for all 4 age groups with the largest decreases in persons aged 20 to 34 years. Rates for firearm suicides increased from 9.32 deaths per 100 000 people in 1989 to 1993 to 10.53 deaths per 100 000 people in 2015 to 2019, but increases were not uniform across age groups (eTable 4 in the Supplement).

**Figure 1.**
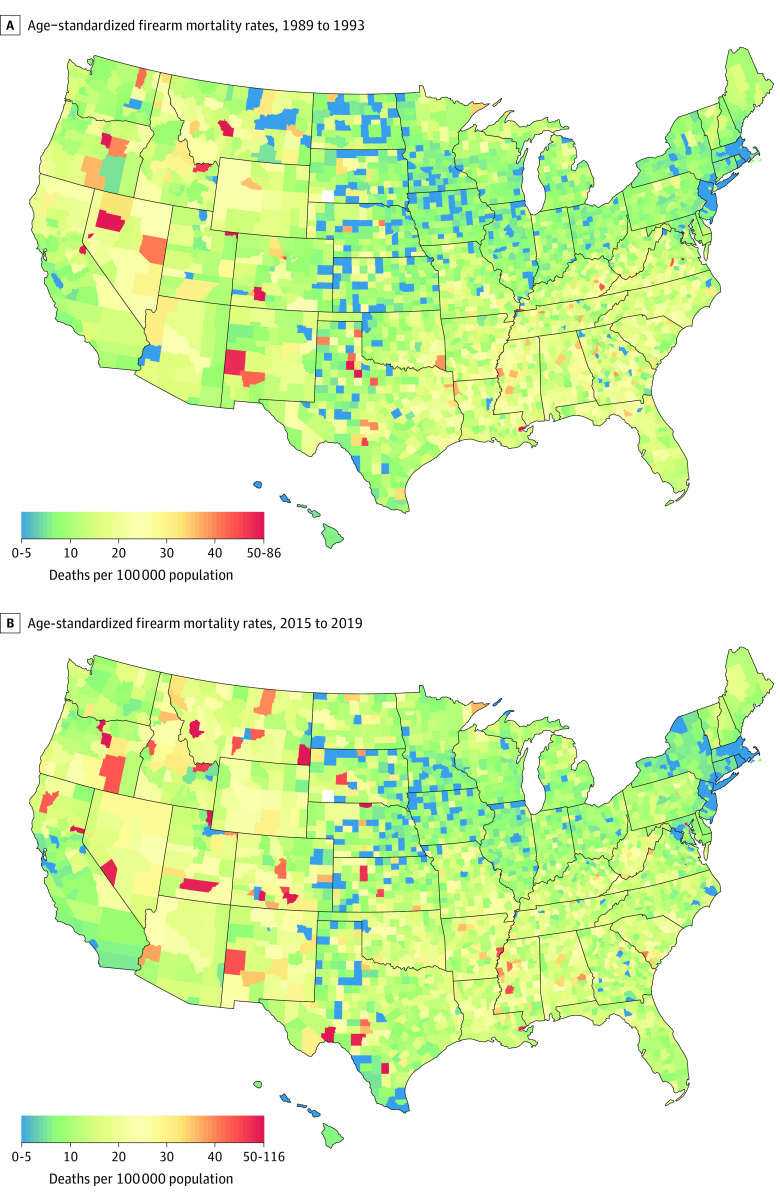
Age-Standardized Firearm Mortality Rates in 1989 to 1993 and 2015 to 2019 Firearm mortality rates include causes of death by homicide, suicide, unintentional death, and of undetermined intent. The top panel shows age-standardized firearm mortality rates in 1989 to 1993. The bottom panel shows age-standardized firearm mortality rates in 2015 to 2019. Alaska is not shown on these maps because it was excluded as a result of definitional inconsistencies during the study period.

Age-standardized firearm mortality rates and changes from 1989 to 1993 vs 2015 to 2019 varied widely among counties (Figure 1). Table 1 summarizes the 25 counties that showed the largest increases and decreases in firearm mortality rates. A disproportionate number of counties in Texas were among those that showed the largest increases and decreases, irrespective of whether the firearm death was a homicide or suicide (eTables 5 and 6 in the Supplement). Of the 25 counties showing the largest decreases over time, District of Columbia had the highest firearm homicide rate of 56.5 homicides per 100 000 people in 1989 to 1993, which then decreased by 4-fold to 14.5 homicides per 100 000 people in 2015 to 2019. In addition, large decreases were seen in 3 counties in New York (Bronx, New York, and Kings County), where rates decreased from approximately 20 homicides per 100 000 people (range, 18.5-23.6 homicides per 100 000 people) to 2 homicides per 100 000 people (range, 1.3-3.5 homicides per 100 000 people) over this 27-year period (eTable 5 in the Supplement). Higher rates of firearm homicides generally clustered in the Southern and Southeastern regions, which further increased over time, especially for Mississippi (eFigure 3 in the Supplement). In contrast, higher rates of firearm suicides were concentrated in more rural regions in the West and Midwest and continued to increase from 1989 to 1993 vs 2015 to 2019 (eFigure 4 in the Supplement). Counties in Nevada and South Dakota, however, showed visible reductions in rates of firearm suicides.

**Table 1.** Largest Decreases and Increases in County-Level Firearm Death Rates, 1989 to 1993 vs 2015 to 2019

Rank	Largest decreases in firearm death rates (per 100 000)						Largest increases in firearm death rates (per 100 000)					
	County name	State	Population size^b^	Age-standardized rate^a^		Rate change, %^c^	County name	State	Population size^b^	Age-standardized rate^a^		Rate change, %^c^
				1989-1993	2015-2019					1989-1993	2015-2019	
1	Hinsdale	Colorado	765	DS	DS	−76.58 (NA)	Terrell	Texas	996	DS	DS	102.79 (802.16)
2	King	Texas	307	DS	DS	−64.26 (NA)	Keya Paha	Nebraska	902	DS	DS	68.95 (NA)
3	Meagher	Montana	1999	DS	DS	−62.11 (−78.14)	McMullen	Texas	883	DS	DS	55.85 (NA)
4	Alpine	California	1159	DS	DS	−49.63 (NA)	Carter	Montana	1320	DS	DS	43.13 (471.95)
5	District of Columbia	DC	55 0521	60.87	15.99	−44.88 (−73.73)	Deer Lodge	Montana	8948	DS	DS	41.69 (405.7)
6	Storey	Nevada	4074	DS	DS	−44.8 (−57.31)	Graham	Kansas	2721	DS	DS	41.14 (629.76)
7	Oldham	Texas	2118	DS	DS	−40.44 (NA)	Harney	Oregon	6898	DS	DS	38.98 (745.21)
8	Pershing	Nevada	6360	DS	DS	−39.32 (−66.69)	Campbell	South Dakota	1565	DS	DS	38.43 (NA)
9	Daggett	Utah	943	DS	DS	−38.15 (−49.39)	La Paz	Arizona	20 238	DS	DS	37.93 (NA)
10	Houston	Tennessee	7988	DS	DS	−36.86 (−93.17)	Blaine	Nebraska	484	DS	DS	36.75 (NA)
11	Cumberland	Virginia	9378	DS	DS	−35.85 (−81.58)	Taliaferro	Georgia	1826	DS	DS	36.05 (NA)
12	Winchester City	Virginia	25 119	56.22	20.55	−35.67 (−63.44)	Huerfano	Colorado	7771	DS	DS	35.8 (226.34)
13	Kenedy	Texas	417	DS	DS	−33.72 (NA)	Haakon	South Dakota	1912	DS	DS	35.29 (342.56)
14	Lafayette	Florida	7953	DS	DS	−33.45 (−94.65)	Foard	Texas	1518	DS	DS	35.21 (NA)
15	Cottle	Texas	1746	DS	DS	−33.32 (NA)	Stafford	Kansas	4488	DS	DS	34.87 (326.49)
16	Keweenaw	Michigan	2195	DS	DS	−33.02 (NA)	Esmeralda	Nevada	787	DS	DS	34.25 (236.76)
17	Wheeler	Oregon	1455	DS	DS	−32.97 (−38.77)	Custer	Colorado	3860	DS	DS	34.01 (297.4)
18	Motley	Texas	1299	DS	DS	−32.6 (−58.16)	Sweet Grass	Montana	3672	DS	DS	32.92 (281.77)
19	Throckmorton	Texas	1618	DS	DS	−32.47 (−74.65)	Rich	Utah	2051	DS	DS	30.5 (132.84)
20	Fulton	Kentucky	7217	DS	DS	−30.35 (−68.06)	Eddy	North Dakota	2626	DS	DS	30.41 (NA)
21	Sanborn	South Dakota	2541	DS	DS	−29.46 (NA)	Loving	Texas	62	DS	DS	29.3 (NA)
22	Sheridan	North Dakota	1430	DS	DS	−28.91 (NA)	Wells	North Dakota	4574	DS	DS	28.6 (NA)
23	Hall	Texas	3700	DS	DS	−28.76 (−68.46)	Sierra	California	3434	DS	DS	27.74 (124.82)
24	Garfield	Nebraska	1816	DS	DS	−28.27 (NA)	Sherman	Oregon	1749	DS	DS	27.63 (91.65)
25	Hyde	North Carolina	5413	DS	DS	−26.93 (−86.4)	Adams	Idaho	3591	DS	DS	27.39 (166.46)

Abbreviations: DS, data suppressed; NA, not applicable.

^a^ Rates based on small counts (<10), or which could be derived from rate change, were suppressed to preserve data confidentiality.

^b^ Population sizes were based on estimates from 2005 (ie, study period midpoint).

^c^ Percentage change is not available for counties that have rates that equal 0. For example, dividing by 0 produces unmeaningful estimates (ie, infinity).

### County Outliers

**Figure 2.**
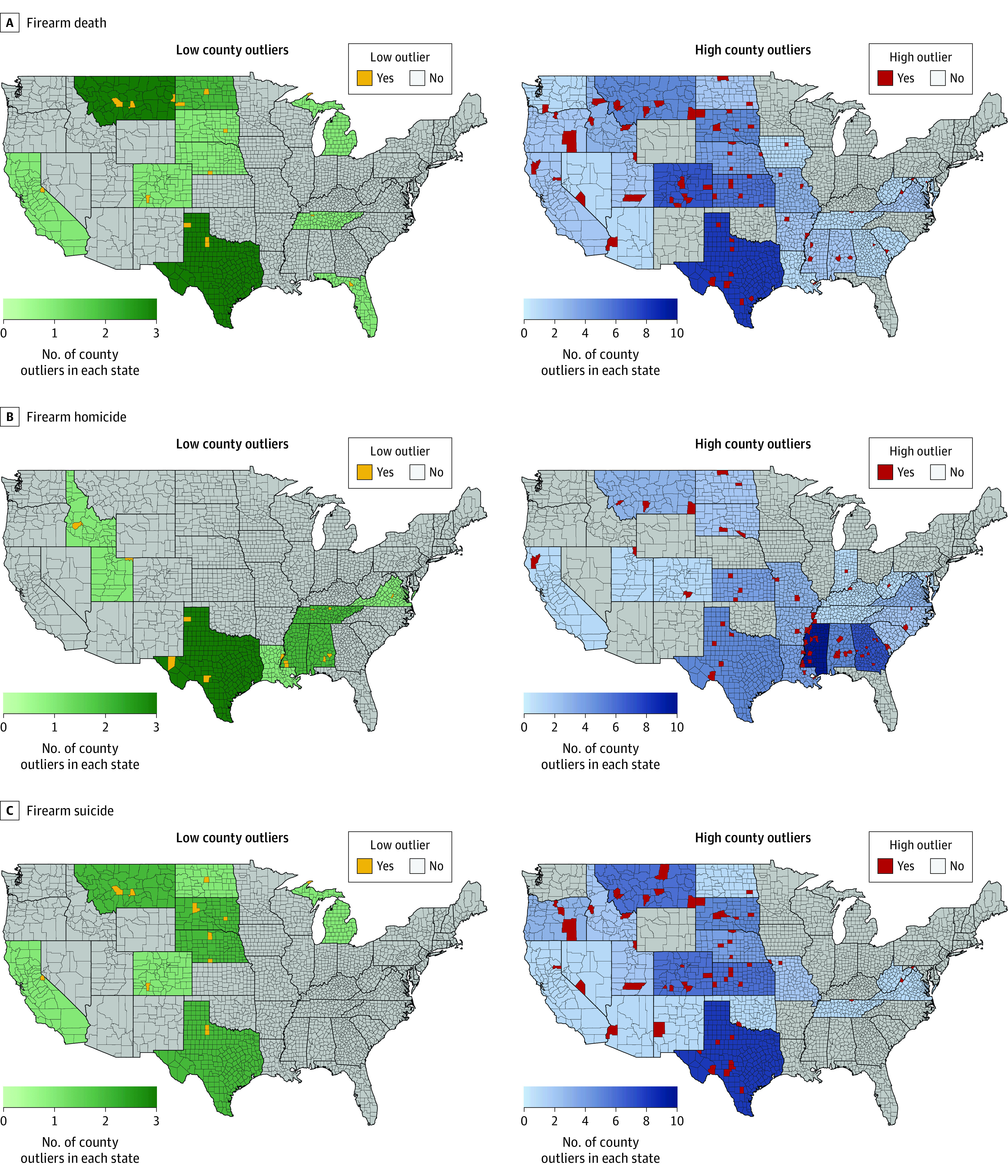
Low and High County Outliers (Hot Spots) for Firearm Mortality Rates in 2015 to 2019, in the Contiguous 48 US States Low county outliers represent below-expected rates, and high county outliers represent above-expected rates, according to previous mortality rates in 1989 to 1993.

More county outliers had above-expected rates than below-expected rates in 2015 to 2019 for firearm death, firearm homicide, and firearm suicide (Figure 2). The preponderance of high county outliers indicates more unexpected increases than unexpected decreases over time. For firearm death, we identified 15 low outliers and 67 high outliers (Table 2). Texas had the highest number of both low and high county outliers. Low county outliers accounted for a small percentage of all firearm deaths in their state (mean [SD], 0.02% [0.04%]) since these counties were very rural and 11 of the 15 counties had zero observed firearm deaths in 2015 to 2019. These low outliers had a small percentage of their population that were Black (only 2.5%) and few federal firearm licenses per capita. By comparison, high county outliers were less rural and included counties that accounted for a disproportionate percentage of their states’ firearm death toll in 2015 to 2019. Baltimore City was responsible for more than half of all deaths in Maryland (1678 deaths [46.8%]), whereas St Louis City, Missouri, and Hinds City, Mississippi, were responsible for approximately 20% (1241 deaths in St. Louis City and 546 deaths in Hinds City) of all firearm deaths in those states.

**Table 2.** Low and High County Outliers for Firearm Death Rates, 2015 to 2019

County name	State	Age-standardized rate, 2015-2019^a^		State firearm deaths, %^d^	County characteristics^b^														
		Observed^c^	Expected (95% CrI)		Population size	RUCC	Land area (square miles)	Sex ratio (M/F)	Population demographics, %				Median household income, $	Population characteristic, %				Trauma care access^f^	Firearm licenses^g^
									Aged 15-65 y	Black	Hispanic	High school graduate		UR^e^	Poverty	Republican voters	Heavy drinkers		
**Low outliers (below-expected rates in 2015-2019)**																			
Furnas	Nebraska	DS	10.64 (4.13-17.2)	0.11	5019	9	718.09	0.91	59.57	0.14	1.28	86.5	33 011	3.7	12	79.04	11.1	6	0
Meagher	Montana	DS	27.04 (20.45-33.69)	0.1	1999	9	2391.82	1	63.98	0	1.55	82.3	29 099	4.3	17	71.74	18.5	11	0
Houston	Tennessee	DS	10.94 (4.26-17.68)	0.02	7988	8	200.21	0.99	63.98	3.34	2.12	79.6	32 590	8.5	18	40.02	13.7	1	1
Lafayette	Florida	DS	8.9 (2.21-15.63)	0.01	7953	8	542.84	1.62	71.37	16.74	11.96	81.3	31 038	3.1	24	73.98	16.7	2	1
Alpine	California	DS	9.71 (3.05-16.44)	0	1159	8	738.62	1.11	74.12	0.69	8.63	92.1	45 283	7.9	17	44.37	25.2	1	0
Hinsdale	Colorado	DS	17.44 (10.53-24.44)	0	765	9	1117.68	1.01	67.71	0	1.57	90.8	42 012	2.8	9	58.97	18	4	0
Keweenaw	Michigan	DS	7.67 (0.99-14.4)	0	2195	9	540.97	1.1	63.87	1.91	0.55	90.8	31 809	10.5	15	54.27	20	1	0
Golden Valley	Montana	DS	9.68 (3.04-16.37)	0	1159	8	1175.3	1.04	65.14	0	1.38	86.7	27 455	4.3	20	75.86	17.5	1	0
Wibaux	Montana	DS	6.84 (0.26-13.46)	0	951	9	889.31	0.95	62.15	0	0.53	75.1	30 663	3.3	13	72.68	11	1	0
Sheridan	North Dakota	DS	9.26 (2.64-15.94)	0	1430	9	971.75	1.03	60.63	0.14	0.35	80.1	29 229	5.9	20	77.01	10.2	0	0
Slope	North Dakota	DS	6.53 (0.01-13.08)	0	709	9	1217.94	1.21	67.56	0	0	91.4	30 729	2.3	12	77.55	9.9	0	0
Sanborn	South Dakota	DS	6.8 (0.28-13.37)	0	2541	9	569.01	1.05	64.46	0	1.26	84.6	35 269	3.4	14	57.29	14.9	1	0
Cottle	Texas	DS	8.22 (1.68-14.8)	0	1746	9	901.18	0.89	57.22	11.8	22.05	79.1	28 011	5.5	21	71.48	11.2	3	0
King	Texas	DS	11.86 (5.21-18.57)	0	307	9	912.29	1.31	71.66	0	15.31	90.8	41 738	4.1	14	87.82	12.8	2	0
Oldham	Texas	DS	9.52 (2.89-16.22)	0	2118	8	1500.63	1.07	64.07	2.83	12.56	82.3	36 521	3.7	14	86.95	11.4	1	0
**High outliers (above-expected rates in 2015-2019)**																			
Baltimore City	Maryland	52.7	42.07 (34.7-49.33)	46.83	635 815	1	80.8	0.87	66.93	64.86	2.25	77.4	32 453	6.9	22	16.96	13.4	-	7
St Louis City	Missouri	77.98	60.09 (52.58-67.46)	19.8	344 362	1	61.92	0.9	66.84	50.68	2.45	80.6	30 629	7.9	26	19.22	15.3	-	3
Orleans Parish	Louisiana	47.88	41 (34.14-47.8)	19.29	454 863	1	180.56	0.89	67.34	67.52	3.12	83.4	30 216	-	26	21.74	11.7	31	1
Hinds	Mississippi	45.89	37.04 (30.45-43.58)	17.39	249 345	2	869.18	0.9	67	65.24	0.91	83.6	35 433	6.7	22	39.97	14	85	0
Deer Lodge	Montana	51.97	39.83 (32.98-46.59)	2.12	8948	7	736.98	1.01	67.58	0.32	2.02	85.1	30 579	5.5	16	37.97	18.3	14	0
Leflore	Mississippi	41.8	34.54 (27.91-41.12)	1.88	36 431	5	591.93	0.92	65.06	70.6	2.18	68.8	22 640	10	37	37.19	14.3	14	1
Bottineau	North Dakota	DS	27.62 (20.78-34.41)	1.82	6741	9	1668.59	1.03	65.63	0.28	0.65	85.9	35 999	4.1	12	67.17	12.9	14	0
Morgan	West Virginia	33.95	25.95 (19.23-32.62)	1.79	16 022	3	228.98	0.99	65.93	0.66	0.84	84.1	40 171	4.5	11	65.88	11.4	8	1
Ransom	North Dakota	DS	23.38 (16.76-29.95)	1.62	5810	8	862.75	1.05	63.58	0.21	1.36	87.3	42 714	3.2	10	51.96	12.1	12	0
Petersburg City	Virginia	50.81	38.58 (31.56-45.51)	1.59	32 604	1	22.88	0.84	62.19	78.57	2.07	72	30 942	7.2	22	18.73	10.5	-	1
Phillips	Arkansas	45.17	36.29 (29.68-42.84)	1.57	24 107	7	692.67	0.85	60.17	61.37	1.51	70.9	24 141	8.9	34	35.65	13.6	11	2
Kingsbury	South Dakota	DS	22.06 (15.46-28.62)	1.54	5532	9	838.37	0.95	60.59	0.04	0.76	87.1	35 604	3.8	11	59.85	11.4	11	0
Clearwater	Idaho	36.04	29.37 (22.82-35.88)	1.4	8373	6	2461.4	1.19	66.57	0.29	1.71	84.7	35 828	9.4	16	70.38	16.1	17	0
Dallas	Alabama	34.31	27.46 (20.84-34.03)	1.29	44 366	4	980.71	0.84	63.67	66.94	0.66	76.8	24 936	7.5	35	39.49	13.2	16	1
Park	Colorado	42.06	33.67 (27.07-40.22)	0.94	16 949	1	2200.69	1.06	74.23	0.76	5.85	93.5	55 589	4.8	8	57.21	15.2	10	2
Burt	Nebraska	DS	18.55 (11.92-25.13)	0.8	7455	8	492.77	0.94	60.47	0.3	1.38	90.4	36 508	5	10	64.04	12.6	3	1
Granite	Montana	DS	49.54 (42.51-56.48)	0.77	2965	8	1727.44	1.03	68.36	0	1.28	90.8	32 063	5.4	15	71.28	18.8	6	0
Sweet Grass	Montana	DS	33.74 (26.91-40.49)	0.77	3672	9	1855.08	1.02	66.07	0.11	1.85	92.8	36 981	1.9	11	76.1	12.2	9	0
Haakon	South Dakota	DS	32.56 (25.81-39.24)	0.77	1912	8	1812.97	0.99	63.18	0	0.78	88	35 183	3.2	11	81.21	12.9	4	0
Garfield	Utah	49.05	39.5 (32.79-46.15)	0.74	4470	9	5174.22	1.06	60.13	0.18	3.13	91.9	38 751	7.2	10	85.48	16.6	7	0
La Paz	Arizona	37.93	26.18 (19.46-32.84)	0.72	20 238	6	4499.95	1.03	54.94	1.01	22.92	76.9	29 015	6.7	22	62.47	19.5	2	0
Franklin	Iowa	21.51	14.94 (8.45-21.38)	0.72	10 732	7	582.44	1	63.29	0.11	10.55	84.3	41 728	4.7	10	56.66	12	5	0
Adams	Idaho	DS	33.82 (27.11-40.46)	0.7	3591	8	1364.58	1.03	67.53	0.08	2.14	88.4	38 028	8.1	14	71.16	15.6	12	0
Musselshell	Montana	DS	35.84 (29.12-42.51)	0.68	4497	8	1867.15	0.94	67.29	0.09	1.89	86.3	30 386	5	18	74.01	15.1	10	0
Macon	Alabama	39.19	31.66 (25.03-38.24)	0.67	22 810	6	610.52	0.86	67.34	82.84	0.96	78.8	23 500	5.1	32	16.69	14.7	8	0
Harney	Oregon	44.22	31.82 (25.1-38.47)	0.62	6898	7	10 134.33	1.05	64.24	0.19	3.51	90.2	33 795	8.8	15	76.04	16.1	14	0
Buffalo	South Dakota	DS	23.21 (16.52-29.86)	0.58	2100	9	470.59	0.99	60.71	0.19	1.19	76.5	16 868	14.8	39	26.52	32.6	0	0
Newton	Arkansas	36.16	29.33 (22.71-35.9)	0.51	8452	9	822.97	1.03	66.56	0.2	1.23	78	27 290	4.9	23	63.48	15.1	5	0
Clear Creek	Colorado	41.28	34.38 (27.67-41.03)	0.51	9197	1	395.45	1.07	75.49	0.41	4.18	96.9	61 937	4.8	7	44.93	15.7	8	2
Stafford	Kansas	DS	33.09 (26.28-39.82)	0.46	4488	9	792.05	0.97	62.32	0.18	7.22	87.7	34 077	3.8	14	75.43	11.1	2	0
Gosper	Nebraska	DS	25.9 (19.08-32.67)	0.45	2020	9	458.18	1.02	60.64	0	1.34	94.4	41 688	3.5	8	79.54	9.7	4	0
Skamania	Washington	31.42	24.48 (17.93-30.98)	0.45	10 664	1	1656.44	1.01	71.16	0.4	4.68	90.2	43 206	7.6	11	52.24	16.4	3	2
Conejos	Colorado	37.09	30.37 (23.74-36.95)	0.41	8512	9	1287.22	0.97	61.24	0.31	55.93	81.4	28 010	7.9	23	49.01	12.4	8	0
Allendale	South Carolina	40.94	32.07 (25.38-38.71)	0.4	10 917	6	408.2	1.11	65.84	72.28	2.26	73.2	22 491	10.6	38	27.43	13.1	3	2
Campbell	South Dakota	DS	27.03 (20.2-33.78)	0.39	1565	9	735.79	1.01	59.62	0	0.26	86	31 652	3.7	12	73.83	10.5	4	0
Harding	South Dakota	DS	24.59 (17.99-31.15)	0.39	1218	9	2670.5	1.05	67.57	0	1.07	90.3	31 327	3.4	14	86.38	11.1	1	0
Huerfano	Colorado	51.62	40.22 (33.42-46.95)	0.38	7771	6	1590.87	1.16	66.18	2.99	34.77	85.1	28 334	7.9	23	49.97	12.7	10	0
Republic	Kansas	DS	23.78 (17.11-30.39)	0.36	5164	9	716.38	0.96	59.35	0.39	1.14	95	31 364	4	10	77.47	12.2	8	0
Keya Paha	Nebraska	DS	43.98 (36.6-51.24)	0.34	902	9	773.29	1	58.43	0	4.66	91	31 082	3.4	19	80.51	9.6	2	0
Rich	Utah	DS	38.48 (31.69-45.18)	0.32	2051	8	1028.53	1.04	64.46	0	1.95	94.9	45 335	3.2	10	88.91	14.8	3	2
Chautauqua	Kansas	DS	28.53 (21.81-35.2)	0.31	4109	9	641.69	0.95	60.5	0.37	1.65	87	32 658	5.2	16	78.01	12.8	4	1
Graham	Kansas	DS	34.53 (27.66-41.31)	0.31	2721	9	898.29	0.98	60.27	3.27	0.85	91.6	33 029	3.4	12	75.14	12.4	5	0
Trego	Kansas	DS	37.68 (30.87-44.42)	0.31	3050	9	888.29	0.91	60	0.39	0.95	88.7	31 258	3.3	13	72.66	12.1	1	0
Custer	Colorado	45.45	36.86 (29.96-43.68)	0.26	3860	8	738.89	1.03	66.87	0.44	3.24	93.7	40 946	4.8	13	68.25	12.5	4	0
Montgomery	Missouri	30.31	22.81 (16.25-29.34)	0.26	12 166	8	537.46	1	64.22	2.15	0.92	78.3	35 093	5.8	14	61.86	14.3	14	2
Clay	Tennessee	35.85	27.67 (21.09-34.21)	0.23	7992	8	236.11	0.97	68.19	1.75	2.3	71.8	25 865	11.5	22	49.15	14.4	3	0
Hamilton	Kansas	DS	24.13 (17.52-30.71)	0.2	2604	9	996.49	0.98	61.94	0.73	26.5	80.9	34 324	3.2	13	78.58	11.6	3	1
Trinity	California	43.77	34.68 (27.9-41.39)	0.19	13 622	8	3178.61	1.05	67.6	0.53	4.77	90.1	31 434	10.2	16	54.66	20.1	15	0
Daviess	Missouri	29.85	22.91 (16.29-29.48)	0.19	8121	8	566.97	0.93	62.82	0	0.94	84	33 940	4.9	17	61.97	13.4	11	4
Carter	Montana	DS	36.81 (29.88-43.65)	0.19	1320	9	3339.57	0.97	67.58	0.08	0.68	91.1	29 496	3.6	13	87.87	13.3	2	0
Sherman	Oregon	DS	44.9 (37.68-52.02)	0.19	1749	9	823.21	1	63.98	0.29	6.52	90	38 806	6.8	16	62.86	17.4	6	0
Clark	Idaho	DS	39.02 (32.32-45.67)	0.16	943	8	1764.63	1.13	61.61	0.11	39.45	68.9	32 687	5	20	85.55	13.8	2	0
Blaine	Nebraska	DS	25.21 (18.46-31.9)	0.11	484	9	710.74	1.08	60.74	0	0.21	95.7	31 533	3.8	18	88.79	9.1	0	0
Cheyenne	Colorado	DS	28.3 (21.58-34.96)	0.08	1953	9	1781.35	0.97	64.11	0.56	8.96	87.9	39 252	3.1	13	81.39	11.8	2	0
Esmeralda	Nevada	DS	36.15 (29.13-43.07)	0.08	787	9	3588.5	1.16	64.68	0.13	11.69	84.1	38 527	4.8	15	76.3	24.6	3	0
Highland	Virginia	DS	29.06 (22.24-35.82)	0.08	2475	9	415.86	0.98	63.52	0.16	0.48	73.7	34 519	3.4	13	64.61	18.5	7	0
Goliad	Texas	30.02	23.33 (16.62-29.99)	0.07	7102	3	853.52	0.99	64.63	5.11	36.22	83.8	38 218	4.8	17	64.75	12.9	5	0
Sierra	California	DS	37.34 (30.43-44.16)	0.05	3434	8	953.38	1.02	67.47	0.26	8.42	88.3	39 380	8.4	11	64.12	21.3	2	0
Mineral	Colorado	DS	50.99 (44.05-57.85)	0.05	932	9	875.72	1.05	65.88	0	2.04	97.4	40 134	5	10	61.87	13.6	1	0
Carson	Texas	DS	24.28 (17.77-30.75)	0.05	6586	3	923.19	0.98	64.17	0.94	8.59	87.9	41 245	3.9	9	83.22	11.3	2	0
Haskell	Texas	DS	21.04 (14.48-27.55)	0.05	5541	6	902.97	0.88	58.85	3.86	23.59	77.9	26 636	3.8	24	63.7	12.8	6	0
Taliaferro	Georgia	DS	26.08 (19.28-32.81)	0.04	1826	8	195.39	0.91	63.64	61.17	1.04	58.4	24 893	7.4	29	35.23	12.2	1	1
Terrell	Texas	DS	79.98 (71.21-88.48)	0.03	996	9	2357.72	0.97	63.55	0	51.1	80.4	27 927	7	20	65.25	9.9	1	0
Edwards	Texas	DS	38.88 (32.22-45.49)	0.02	1987	9	2119.75	1.05	67.44	3.02	46.15	67.7	27 942	3.9	29	77.36	15.6	1	0
Menard	Texas	DS	34.93 (28.37-41.45)	0.02	2201	8	901.91	1.01	61.38	1.14	31.53	80.1	27 013	4.5	26	68.99	14.7	5	0
Foard	Texas	DS	24.17 (17.45-30.83)	0.01	1518	9	706.68	0.92	58.1	3.62	18.38	75.8	25 535	4.7	18	59.11	10.3	1	0
McMullen	Texas	DS	37.49 (30.28-44.57)	0.01	883	8	1113	1.05	68.86	1.13	35.33	78.7	36 046	5.2	15	82.8	15.4	1	2

Abbreviations: CrI, credible intervals; DS, data suppressed; RUCC, rural-urban continuum code; UR, unemployment rate.

^a^ Expected age-standardized mortality rates were estimated from our bayesian models for all 3111 counties.

^b^ See eTable 1 in the Supplement for details on county characteristics. Timings of measures range from 1999 to 2010, with most characteristics measured in 2005.

^c^ Rates based on small counts (<10), or which could be derived from rate change, were suppressed to preserve data confidentiality.

^d^ The percentage of state firearm deaths that occured in rural counties in 2015 to 2019 accounted for by that county outlier.

^e^ Refers to umber unemployed as a percentage of the labor force; see eTable 1 in the Supplement.

^f^ Number of level 1 trauma centers within 60 miles (direct distance).

^g^ Per capita prevalence of type 1 (firearm dealer) and type 2 (pawnbroker) federal firearm licenses.

We identified 14 low and 63 high county outliers for firearm homicide (eTable 7 in the Supplement). District of Columbia showed a large, unexpected decrease in firearm homicide rates, although observed rates remained fairly high in 2015 to 2019 at 14.5 homicides per 100 000 people. Although Texas had a similarly high number of low outliers for firearm homicide, as with firearm death, all identified counties in Texas accounted for less than 0.05% of the state’s homicides. High outliers, however, made up a substantial proportion of state firearm homicide. Twelve high outlier counties were responsible for more than 10% (range, 10.6%-66.7%) of all firearm homicides in their state, even though their population sizes accounted for a smaller proportion of their state’s total population (range, 4.9%-16.8%). For example, the firearm homicide rate in Baltimore City almost doubled, from 29.71 to 47.43 per 100 000. Baltimore City was again responsible for more than half (66.7%) of firearm homicides in Maryland (1510 homicides), despite only accounting for 11.4% of the state’s population. Most high outlier counties were in the Southeast, notably Alabama, Mississippi, and Georgia. The high county outliers in Alabama accounted for almost half of its state homicides in 2015 to 2019, with 31.4% of its firearm homicides (721 homicides) occurring in Jefferson County and 10.6% (243 homicides) occurring in Montgomery County. High county outliers were more urban and educated yet with higher unemployment rates and a higher percentage living in poverty compared with low county outliers. In addition, high county outliers had, on average, 5-fold more federal firearm licenses per capita.

For firearm suicide, we identified 12 low outliers and 53 high outliers (eTable 8 in the Supplement), and most high outliers were concentrated in counties in the West and Midwest. Although Texas and Montana had the highest number of low and high county outliers, no single county outlier (low or high) accounted for more than 2% of its state firearm suicide toll in 2015 to 2019. Low and high outliers were extremely rural and only small percentages of their populations were Black or Hispanic.

### County Outlier Comparisons

Table 3 and eTable 9 in the Supplement show comparisons of characteristics between counties that were not identified as outliers and low and high outliers. County characteristics significantly varied by outlier status, as well as by cause of firearm death. For unexpected changes in firearm homicide, sociodemographics, economy, politics, and the prevalence of federal firearm licenses emerged as important characteristics. Compared with counties that were not outliers, counties that showed unexpected increases in firearm homicides were characterized by more women, a higher percentage of people who are Black, and a smaller percentage of persons aged 15 to 65 years and who voted Republican in 2004. These high county outliers were also poorer, with a higher percentage of unemployment and poverty and a lower median household income, and had more federal firearm licensed dealers per capita.

**Table 3.** Comparisons of County Characteristics by Outlier Status (No Outlier, Low Outlier, High Outlier)

Characteristic^a^	Firearm death				Firearm homicide				Firearm suicide			
	Mean (SD)				Mean (SD)				Mean (SD)			
	No outlier (n = 3029)	Low outlier (n = 15)	High outlier (n = 67)	*P* value^b^	No outlier (n = 3034)	Low outlier (n = 14)	High outlier (n = 63)	*P* value^b^	No outlier (n = 3046)	Low outlier (n = 12)	High outlier (n = 53)	*P* value^b^
Geography												
RUCC	5.05 (2.66)	8.67 (0.49)	7.01 (2.67)	<.001	5.10 (2.67)	6.71 (3.02)	5.11 (3.02)	.079	5.04 (2.66)	8.83 (0.39)	8.13 (1.53)	<.001
Land area, square miles	944.76 (1300.86)	959.18 (512.58)	1334.57 (1488.67)	<.001	957.17 (1314.71)	1002.42 (1019.95)	751.95 (639.92)	.7	940.55 (1292.12)	1101.69 (555.57)	1647.50 (1810.42)	<.001
Sociodemographic												
Sex ratio (M/F)	0.99 (0.09)	1.09 (0.18)	0.99 (0.07)	.003	0.99 (0.09)	1.01 (0.11)	0.94 (0.07)	<.001	0.99 (0.09)	1.03 (0.11)	1.01 (0.05)	<.001
Aged 15-65 y, %	66.07 (3.26)	65.17 (4.65)	64.30 (3.72)	<.001	66.05 (3.30)	66.44 (2.30)	64.79 (2.59)	.023	66.06 (3.25)	64.44 (4.91)	64.25 (4.14)	<.001
Black, %	9.03 (14.30)	2.51 (4.99)	11.67 (25.17)	<.001	8.44 (13.57)	23.88 (25.54)	35.48 (27.47)	<.001	9.23 (14.69)	1.22 (3.38)	0.64 (1.33)	<.001
Hispanic, %	7.09 (12.56)	5.41 (6.93)	8.55 (13.61)	.6	7.12 (12.55)	11.74 (20.83)	5.64 (10.20)	.5	7.07 (12.56)	4.67 (7.04)	9.73 (13.11)	.3
Education												
High school graduate, %	83.04 (7.33)	84.90 (5.42)	84.10 (8.08)	.3	83.18 (7.30)	75.96 (7.49)	79.45 (7.45)	<.001	83.01 (7.34)	85.75 (5.16)	86.26 (7.13)	.002
Economic												
Median household income, $	39 209.42 (10 066.57)	33 630.47 (5484.75)	33 683.72 (7317.72)	<.001	39 258.12 (10 039.63)	31 921.36 (7169.45)	31 278.94 (5987.54)	<.001	39 168.94 (10 069.29)	33 035.00 (7003.28)	34 369.85 (6701.12)	<.001
Unemployment rate, %^c^	5.37 (1.73)	4.89 (2.36)	5.70 (2.44)	.2	5.34 (1.72)	6.56 (2.82)	6.86 (2.44)	<.001	5.38 (1.74)	5.29 (2.34)	5.12 (2.42)	.06
Poverty, %	15.33 (6.50)	16.00 (4.05)	17.60 (7.87)	.10	15.20 (6.38)	21.64 (8.80)	22.83 (8.08)	<.001	15.36 (6.51)	18.58 (10.82)	15.83 (6.11)	.4
Politics												
Republican voters, %	60.18 (12.35)	68.60 (14.42)	61.56 (19.61)	.01	60.48 (12.31)	55.82 (20.80)	50.08 (17.63)	<.001	60.04 (12.46)	66.68 (15.35)	70.55 (13.48)	<.001
Health												
Heavy drinkers, %	13.15 (2.28)	14.81 (4.42)	14.16 (3.70)	.11	13.17 (2.34)	14.25 (1.72)	13.21 (2.17)	.079	13.15 (2.26)	16.62 (5.99)	14.35 (4.07)	.045
Trauma care access^d^	26.47 (43.67)	2.33 (2.87)	7.69 (11.28)	<.001	1.45 (2.87)	0.64 (1.65)	0.94 (1.27)	.092	1.46 (2.87)	0.00 (0.00)	0.25 (0.73)	<.001
Firearm dealers												
Firearm licenses^e^	1.46 (2.87)	0.13 (0.35)	0.58 (1.18)	<.001	26.05 (43.39)	4.57 (3.55)	26.54 (40.04)	<.001	26.42 (43.59)	3.17 (3.30)	4.98 (4.18)	<.001

Abbreviation: RUCC, rural-urban continuum code.

^a^ See eTable 1 in the Supplement for details on county characteristics. Timings of measures range from 1999 to 2010, with most characteristics measured in 2005.

^b^ Group comparisons across the 3 groups (no outlier, low outlier, high outlier) were performed using Kruskal-Wallis rank sum test. All *P* values were adjusted for multiple testing by using the Benjamini-Hochberg method to control for the false discovery rate. See eTable 9 in the Supplement for posthoc pairwise comparisons.

^c^ Refers to the number unemployed as a percentage of the labor force (eTable 1 in the Supplement).

^d^ Refers to number of level I trauma centers within 60 miles (direct distance).

^e^ Per capita prevalence of type 1 (firearm dealer) and type 2 (pawnbroker) federal firearm licenses.

On the other hand, high county outliers that showed unexpected increases in firearm suicide were more rural with greater land area, had more men, a smaller percentage of people who are Black, and fewer level I trauma care centers in the vicinity. There were, however, minimal differences between low and high outlier counties, irrespective of cause of firearm death. The number of firearm licenses was the only key differentiating characteristic, where high county outliers for firearm deaths and homicide had more federal firearm licensed dealers per capita compared with low county outliers. High outliers for total firearm deaths were also more urban with more women than low outliers (eTable 9 in the Supplement).

## Discussion

This cross-sectional study advances firearm research by using an inductive approach to model county-level variation over 3 decades and identify geographical hot spots in changes in firearm mortality rates. First, we highlight important differences between counties and regions by cause of firearm death (suicide and homicide), which became more exacerbated over time. Second, we identify a handful of geographical hot spots at the county (Baltimore City, Maryland), state (Alabama, Texas, District of Columbia), and regional (Southeast) levels, each providing insights on firearm violence and in need of further investigation. Finally, our study pilots the use of an inductive hot-spotting approach to county-level firearm fatalities and shows that, in cases where fatalities are not highly concentrated to specific geographical regions (eg, firearm suicides), statistical outliers alone may not be sufficient for guiding policy responses. Future prevention efforts could now be targeted to county outliers that substantially contribute to the firearm death toll to effectively tackle the public health burden of firearm violence in the US.

Previous studies^9,12,13,50,51,52,53^ show important geographical differences in firearm mortality rates and trends between states, the urban-rural continuum, and several case-selected smaller geographical units (eg, cities). Alternatively in this study, we examined geographical variation across all US counties. Total firearm deaths increased from 1989 to 1993 vs 2015 to 2019, which was driven by rising rates of firearm suicide. Higher rates of suicide were concentrated in more rural regions in the West and Midwest in 1989 to 1993, regions that then experienced more pronounced increases over time.^54,55^ Although firearm homicide rates decreased during the study period, Southeastern regions that had higher rates in 1989 to 1993 showed localized increases.^56^ Our findings suggest that disparities between counties and regions may be becoming more exacerbated over time. High risk counties and regions continue to experience rising firearm mortality rates, even when national trends show overall reductions (eg, firearm homicide). This underlines the importance of examining spatial patterns locally for identifying increasingly vulnerable populations and the urgency of policy makers to deliver local interventions so not to further exacerbate health disparities.

By adopting a novel inductive approach, we further identified county outliers that were hot spots showing unexpected decreases and increases in firearm mortality rates.^30^ District of Columbia showed significant, unexpected improvements in firearm homicides from 1989 to 1993 vs 2015 to 2019. This large decrease in firearm violence has been noted elsewhere^57^ and may be associated with the adoption of more restrictive licensing of handguns,^58^ the subsiding crack cocaine epidemic,^59^ and the changing population demographic (growth and gentrification).^60^ By contrast, we also observed geographical hot spots for increases in firearm homicides despite the decreasing national trend. For example, age-standardized rates for firearm homicide almost doubled in Baltimore City from 1989 to 1993 vs 2015 to 2019, and by 2015 to 2019, the county had 67% of total firearm homicides in Maryland. More generally, we found that high county outliers for firearm homicide clustered in the Southeast, including Alabama, Mississippi, and Georgia. These high county outliers, particularly urban counties, bore a disproportionate burden of firearm homicides in 2015 to 2019. In Alabama the high county outliers were responsible for almost half of state firearm homicide, with the most urban counties of Jefferson and Montgomery County accounting for 31% and 11%, respectively.

We identified key differences in the characteristics of low and high county outliers, such as poverty,^5,55^ race,^61^ urbanicity,^10^ and federal firearm licensed dealers.^55,62^ Counties that showed unexpected increases in firearm homicide over time were poorer with high unemployment rates and poverty, as well as low median household incomes.^5,55^ High county outliers for firearm homicide also had a large percentage of the population who are Black (35%), compared with counties that were not outliers (8%). Firearm homicide disproportionately affects Black communities,^61^ and firearm incidents are often geographically concentrated in high poverty neighborhoods owing to a history of structural racism and residential segregation.^63^ The finding that high county outliers are characterized by both poverty and a large Black share of the population suggests that the consequences of historical and structural racism are continuing to endure on these marginalized communities.^64^

On the other hand, firearm suicide is documented to disproportionately affect White men and rural communities.^10,61^ In line with this, we found that counties with unexpected increases in firearm suicides were predominantly White (<1% Black share of the population) and rural, with large land areas with poor access to level I trauma care centers. This clustering of characteristics further underlines the importance of firearm suicide as a public health problem among rural counties and the vulnerability of these communities due to a lack of access to health services.^10,65^ Although there were few differences between low and high county outliers, more federal firearm licenses per capita were seen in high county outliers for total firearm death and firearm homicide. This association between federal firearm licenses and firearm homicide rates has been reported elsewhere,^25^ which suggests that licensed firearm dealers could be a modifiable factor associated with risk for future policies that may be reduced to prevent deteriorations in firearm violence. Future in-depth investigations are needed to unpack which characteristics are making communities more vulnerable and driving marked discrepancies between counties. An inductive approach that uses county outliers as the unit of analysis, rather than states or variables, offers the unique opportunity to advance understanding of interacting county and population characteristics that are associated with lethal firearm violence. Importantly, this approach will also enable future studies to investigate whether any effects of policies introduced in a given state over the past 2 decades were uniform or varied from county to county.

Texas had a high number of county outliers for firearm homicide and suicide, for both unexpected decreases (eg, King County) and increases (eg, Terrell County). However, each outlier in Texas accounted for less than 1% of firearm deaths because they were extremely rural with very low (often 0) firearm deaths. Therefore, although Texas appears to have the most within-state variation in unexpected changes over time, this may reflect a statistical bias where counties with few firearm deaths experience significant changes when, in reality, such changes minimally impact the national burden of firearm deaths. This highlights the importance of not only considering statistical outliers, but also considering the number and proportion of firearm deaths that occur in the county. Only then can the most meaningful geographical hot spots be identified and targeted as a priority for future intervention. Unlike for firearm homicide, we failed to detect meaningful hot spots for changes in firearm suicides as its sparse geographical distribution meant that the outliers identified here accounted for too few suicides to justify targeting these areas. Future research should aim to exploit the critical hot spots identified here for firearm homicide and build on our findings to determine whether similarly critical hot spots exist for firearm suicide.

### Limitations

This study has limitations. First, although we argue it is important to maximize the period of potential change to allow future investigations into firearm policies (see Methods, Firearm Mortality Rates subsection), the use of 1989 to 1993 as a baseline period may produce nongeneralizable findings given the national peak in firearm violence during this period.^70^ Second, at the time of analysis, 2019 was the latest available year. This end year will have also impacted our findings, particularly for firearm homicide, as there is now clear evidence that firearm violence has increased since 2019 and during the COVID-19 pandemic period.^66,67^ This study cannot speak to which counties experienced further deteriorations in firearm homicide during the COVID-19 pandemic, but emerging evidence suggests that there may be some overlap, with Baltimore City continuing to show deteriorations in firearm violence in 2020 and 2021.^68,69^ Third, the population estimates, firearm deaths, and county characteristics used are subject to error. Data on county-level characteristics may also be overly restrictive (eg, focusing on access to level I trauma care centers only^35^). Fourth, the bayesian models used in this analysis smooth rates by state and neighboring counties and may therefore attenuate unusually low or high firearm mortality rates in some cases, underestimating true geographical variability. Fifth, our analyses aimed to identify and describe counties that were low and high outliers with respect to changes in firearm mortality rates over time. Future research is needed to explain why these counties are outliers, including determining the role of the county characteristics and why counties within in the same state often respond differently to the introduction of firearm policies.

## Conclusions

Firearm violence is increasingly recognized as an urgent public health problem in the US. Yet, there has been limited progress in preventing avoidable firearm deaths. There is still inconsistent evidence on whether local characteristics and firearm policies explain geographical variation in firearm mortality rates. To alleviate the public health problem of firearm deaths, research and policy should look to these county outliers to learn what has worked and to develop targeted local interventions for the most vulnerable communities in the US.

## Notes

Supplement. **eMethods.** Bayesian Spatial Model **eTable 1.** Outcomes and Corresponding Selected Cause of Death Group (*ICD-9* and *ICD-10* Codes) **eTable 2.** County-Level Characteristics, Timings and Data Sources **eTable 3.** Total Counts of Firearm Deaths by Age Group, 1989 to 2019 **eTable 4.** Mean Changes in Firearm Death Rates from 1989 to 1993 vs 2015 to 2019 **eTable 5.** Largest Decreases and Increases in County-Level Firearm Homicide Rates, 1989 to 1993 vs 2015 to 2019 **eTable 6.** Largest Decreases and Increases in County-Level Firearm Suicide Rates, 1989 to 1993 vs 2015 to 2019 **eTable 7.** Low and High Outliers for Firearm Homicide Rates, 2015 to 2019 **eTable 8.** Low and High Outliers for Firearm Suicide Rates, 2015 to 2019 **eTable 9.** Post Hoc Pairwise Comparisons of County Characteristics by Outlier Status (No Outlier, Low Outlier, High Outlier) **eFigure 1.** Schematic Diagram Illustrating How the Association Between Previous and Recent Firearm Deaths Can Capture Expected (*Top*) and Unexpected (*Bottom*) Change Over Time **eFigure 2.** Schematic Directed Acyclical Graph (DAG) of the Associations Between County-Level Covariates and Previous (1989 to 1993) and Recent (2013 to 2019) Firearm Deaths **eFigure 3.** Age-Standardized Firearm Homicide Rates in 1989 to 1993 and 2015 to 2019 **eFigure 4.** Age-Standardized Firearm Suicide Rates in 1989 to 1993 and 2015 to 2019
eReferences
