# Supplementary material for: County-Level Variation in Changes in Firearm Mortality Rates Across the US, 1989 to 1993 vs 2015 to 2019
Source: JAMA Netw Open. 2022 Jun 6;5(6):e2215557. doi: 10.1001/jamanetworkopen.2022.15557 (PMC9171565; doi:10.1001/jamanetworkopen.2022.15557)
Supplement: Supplement. — eMethods. Bayesian Spatial Model eTable 1. Outcomes and Corresponding Selected Cause of Death Group (ICD-9 and ICD-10 Codes) eTable 2. County-Level Characteristics, Timings and Data Sources eTable 3. Total Counts of Firearm Deaths by Age Group, 1989 to 2019 eTable 4. Mean Changes in Firearm Death Rates from 1989 to 1993 vs 2015 to 2019 eTable 5. Largest Decreases and Increases in County-Level Firearm Homicide Rates, 1989 to 1993 vs 2015 to 2019 eTable 6. Largest Decreases and Increases in County-Level Firearm Suicide Rates, 1989 to 1993 vs 2015 to 2019 eTable 7. Low and High Outliers for Firearm Homicide Rates, 2015 to 2019 eTable 8. Low and High Outliers for Firearm Suicide Rates, 2015 to 2019 eTable 9. Post Hoc Pairwise Comparisons of County Characteristics by Outlier Status (No Outlier, Low Outlier, High Outlier) eFigure 1. Schematic Diagram Illustrating How the Association Between Previous and Recent Firearm Deaths Can Capture Expected (Top) and Unexpected (Bottom) Change Over Time eFigure 2. Schematic Directed Acyclical Graph (DAG) of the Associations Between County-Level Covariates and Previous (1989 to 1993) and Recent (2013 to 2019) Firearm Deaths eFigure 3. Age-Standardized Firearm Homicide Rates in 1989 to 1993 and 2015 to 2019 eFigure 4. Age-Standardized Firearm Suicide Rates in 1989 to 1993 and 2015 to 2019 eReferences [file jamanetwopen-e2215557-s001.pdf]

Supplemental Online Content

Degli Esposti M, Gravel J, Kaufman EJ, Delgado MK, Richmond TS, Wiebe DJ. County-level variation in changes in firearm mortality rates across the US, 1989 to 1993 vs 2015 to 2019. *JAMA Netw Open*. 2022;5(6):e2215557. doi:10.1001/jamanetworkopen.2022.15557

eMethods. Bayesian Spatial Model
eTable 1. Outcomes and Corresponding Selected Cause of Death Group (*ICD-9* and *ICD-10* Codes)
eTable 2. County-Level Characteristics, Timings and Data Sources
eTable 3. Total Counts of Firearm Deaths by Age Group, 1989 to 2019
eTable 4. Mean Changes in Firearm Death Rates from 1989 to 1993 vs 2015 to 2019
eTable 5. Largest Decreases and Increases in County-Level Firearm Homicide Rates, 1989 to 1993 vs 2015 to 2019
eTable 6. Largest Decreases and Increases in County-Level Firearm Suicide Rates, 1989 to 1993 vs 2015 to 2019
eTable 7. Low and High Outliers for Firearm Homicide Rates, 2015 to 2019
eTable 8. Low and High Outliers for Firearm Suicide Rates, 2015 to 2019
eTable 9. Post Hoc Pairwise Comparisons of County Characteristics by Outlier Status (No Outlier, Low Outlier, High Outlier)
eFigure 1. Schematic Diagram Illustrating How the Association Between Previous and Recent Firearm Deaths Can Capture Expected (*Top*) and Unexpected (*Bottom*) Change Over Time
eFigure 2. Schematic Directed Acyclical Graph (DAG) of the Associations Between County-Level Covariates and Previous (1989 to 1993) and Recent (2013 to 2019) Firearm Deaths
eFigure 3. Age-Standardized Firearm Homicide Rates in 1989 to 1993 and 2015 to 2019
eFigure 4. Age-Standardized Firearm Suicide Rates in 1989 to 1993 and 2015 to 2019
eReferences

This supplemental material has been provided by the authors to give readers additional information about their work.

© 2022 Degli Esposti MD et al. *JAMA Network Open.*

eMethods

Bayesian Spatial Model

Our Bayesian hierarchical (spatial) model was specified as:

$$m_i = \beta_0 + \beta_1 X_i + \gamma_i$$

where m_i indicates age-standardised firearm mortality rates for January 1, 2015, to December 31, 2019, for a given county (i), and modelled as a gaussian distribution. The model for m_i contains three main components: an intercept (β_0); fixed covariate effect (β_1); and random spatial effect (γ_i). The model included previous age-standardised firearm mortality rates January 1, 1989, to December 31, 1993, for each county as the covariate (X_i; see methods in the manuscript and eFigure 1for more details). The random spatial effect (γ_1) was assumed to follow intrinsic conditional autoregressive (CAR) prior distribution to account for spatial autocorrelation and smooth over adjacent counties (γ_{1i}) as proposed by Besag.[1,2]

We set the prior on the precision to a Gamma distribution with parameters α=0.001 and β=0.001 as this is a commonly used vague prior. Models were fitted using the Integrated Nested Laplace Approximation method in R (version 4.1.0) (R-INLA); a novel approach to performing approximate Bayesian inference in latent Gaussian models, including generalized linear mixed models and spatial models.[3,4] Integrated Nested Laplace Approximation (INLA) is an alternative to standard Markov Chain Monte Carlo (MCMC) methods for estimating the integral of a posterior (probability) distribution. Whereas MCMC samples from the posterior distribution of model parameters, INLA uses Laplace approximation to estimate the model parameters' posterior marginal distributions reducing the computational time of Bayesian inference.[3,4]

© 2022 Degli Esposti MD et al. *JAMA Network Open.*

eTable 1. Outcomes and Corresponding Selected Cause of Death Group (ICD-9 and ICD-10 codes)

Outcome	Cause of death group	ICD name	ICD-9 codes	ICD-10 codes
Firearm homicide	128	Assault (homicide) by discharge of firearms	E965.0 – E965.4	X93 – X95
Firearm suicide	125	Intentional self-harm (suicide) by discharge of firearms	E955.0 – E955.4	X72 – X74
Unintentional firearm death	119	Accidental discharge of firearms	E992.0 – E992.9	W32 – W34
Undetermined intent of firearm death	132	Discharge of firearms, undetermined intent	35500, 35600	Y22 – Y24

Total firearm deaths in this analysis includes deaths by discharge of firearms due to homicide, suicide, unintentional and undermined intent.

© 2022 Degli Esposti MD et al. *JAMA Network Open.*

eTable 2. County-Level Characteristics, Timings and Data Sources

County-level characteristic	Variables or description	Time period	Data source
**Geography**			
Rural-Urban Continuum Code	Rural-Urban Continuum Code [RUCC range: 1-9].<br><br>Counties with a RUCC score of ≤3 are metropolitan counties and classified as urban; whereas counties with a score of ≥ 4 are non-metropolitan counties and classified as rural (see eTable 3).	2003	2003: U.S. Department of Agriculture. Economic Research Service. 2003 Rural-Urban Continuum Codes. August 2003. http://www.ers.usda.gov/Data/RuralUrbanContinuumCodes/2003/RuralUrbanCodes2003.xls
Land area	Land area in square miles	2000	U.S. Dept. of Commerce, Bureau of the Census, and Inter-university Consortium for Political and Social Research. Census of Population and Housing, 2000 [United States]: Selected Subsets from Summary File 3 [Computer file]. ICPSR13402-v2. Washington, DC: U.S. Dept. of Commerce, Bureau of the Census, and Ann Arbor, MI: Inter-university Consortium for Political and Social Research [producers], 2004.<br>Ann Arbor, MI: Inter-university Consortium for Political and Social Research[distributor], 2004. DS 12 (Counties, Tables P1-P160I Only).
**Sociodemographic**			
Age distribution	Percent of resident population who are aged 15 to 65years	2005	U.S. Department of Commerce, Bureau of the Census. Population Estimates Program. CC-EST2005-agesex-[ST_FIPS]: Annual Estimates of the Population by Selected Age Groups and Sex for Counties in [STATE]: April 1, 2000 to July 1, 2005. August 4, 2006. http://www.census.gov/popest/counties/asrh/CC-EST2005-agesex.html (One data file per state)
Sex distribution	Sex ratio: Male-to-female	2005	U.S. Department of Commerce, Bureau of the Census. Population Estimates Program. CC-EST2005-agesex-[ST_FIPS]: Annual Estimates of the Population by Selected Age Groups and Sex for Counties in [STATE]: April 1, 2000 to July 1, 2005. August 4, 2006. http://www.census.gov/popest/counties/asrh/CC-EST2005-agesex.html (One data file per state)
Race distribution	Percent of resident population who are Black alone	2005	U.S. Department of Commerce, Bureau of the Census, Population Estimates Program. CC-EST2005-6RACE-[ST_FIPS]: County Population Estimates with Sex, 6 Race Groups (5 Race Alone and One Group with Two or More Race Groups) and Hispanic Origin. August 4, 2006. http://www.census.gov/popest/counties/asrh/CC-EST2005-RACE6.html (One data file per state)
Ethnicity distribution	Percent of resident population who are Hispanic (of all races)	2005	U.S. Department of Commerce, Bureau of the Census, Population Estimates Program. CC-EST2005-6RACE-[ST_FIPS]: County Population Estimates with Sex, 6 Race Groups (5 Race Alone and One Group with Two or More Race Groups) and Hispanic Origin. August 4, 2006.

© 2022 Degli Esposti MD et al. *JAMA Network Open.*

County-level characteristic	Variables or description	Time period	Data source
			http://www.census.gov/popest/counties/asrh/CC-EST2005-RACE6.html (One data file per state)
**Education**			
Education in the population	Percent of adults who are from high school graduates or higher	2006-2010 (5-year estimate)	American Community Survey: Educational attainment. TableID: S1501; 2010: ACS 5-Year Estimates Subject Tables https://www.census.gov/data/developers/data-sets/acs-5year.2010.html
**Economics**			
Unemployment rate	Number unemployed as a percent of the labor force	2005	U.S. Department of Labor. Bureau of Labor Statistics. Local Area Unemployment Statistics (LAUS) Program. Labor Force Data by County, 2005 Annual Averages. No date given. Accessed September 11, 2007. ftp://ftp.bls.gov/pub/special.requests/la/laucnty05.txt
Poverty	Percent of people (all ages) living in poverty	2005	U.S. Department of Commerce, Bureau of the Census, Small Area Income and Poverty Estimates (SAIPE) Program. https://www.census.gov/programs-surveys/saipe.html
Household income	Median household income ($)	2005	U.S. Department of Commerce, Bureau of the Census, Small Area Income and Poverty Estimates (SAIPE) Program. https://www.census.gov/programs-surveys/saipe.html
**Politics**			
Republican Voters	Percent of voters who voted for Bush [Republican] in the 2004 presidential election [Bush vs Kerry]	2004	Charles Haines Stewart III. Official 2004 Presidential Election Returns. No date given. Accessed June 18, 2007. http://web.mit.edu/cstewart/www/nationwide2004.xls
**Health**			
Alcohol consumption	Percent of all drinkers who are heavy drinkers	2005	Institute for Health Metrics and Evaluation (IHME). United States Alcohol Use Prevalence by County 2002-2012. Seattle, United States: Institute for Health Metrics and Evaluation (IHME), 2015. http://ghdx.healthdata.org/record/ihme-data/united-states-alcohol-use-prevalence-county-2002-2012 https://vizhub.healthdata.org/subnational/usa
Access to trauma centers	Number of Level 1 Trauma Centers within 60 miles (crow-fly distance)	2005	American Trauma Society. Trauma Information Exchange Program. Available at: http://www.amtrauma.org/tiep/index.html
**Firearm dealers**			
Federal firearm licenses (FFLs)	Per capita prevalence (number) of "type one" (firearm dealer) and "type two" (pawnbroker) FFLs	1999	Bureau of Alcohol, Tobacco, Firearms and Explosives (ATF). Federal Firearms Licenses. Available at: https://www.atf.gov/firearms

© 2022 Degli Esposti MD et al. *JAMA Network Open*.

eTable 3. Total Counts of Firearm Deaths by Age Group, 1989 to 2019

	Study period, 31y (1989-2019), No. (% of total deaths)[a]				
	**All ages**	**0-19y**	**20-34y**	**35-54y**	**55y+**
**Total firearm deaths**	1036518	110640 (10.7)	359965 (34.7)	307320 (29.6)	258593 (24.9)
Firearm homicide	412231 (39.8)	68240 (61.7)	211009 (58.6)	105032 (34.2)	27950 (10.8)
Firearm suicide	589285 (56.9)	33135 (30)	137587 (38.2)	193833 (63.1)	224730 (86.9)
Firearm unintentional deaths[b]	25428 (2.5)	7428 (6.7)	7903 (2.2)	5903 (1.9)	4194 (1.6)
Firearm undetermined intent[b]	9574 (0.9)	1837 (1.7)	3466 (1)	2552 (0.8)	1719 (0.7)

Abbreviation: y, years.
[a] Figures are based on 3111 US counties and complete records which had information on the age of the deceased. Therefore, firearm death cases that had missing age information were excluded (n=1033, less than 0.1% of all records).
[b] Because unintentional deaths and undetermined intent made up fewer than 3% and 1% of all firearm deaths (respectively), and suffered from sparse data, we did not separately analyse these causes of firearm death.

© 2022 Degli Esposti MD et al. *JAMA Network Open.*

eTable 4. Mean Changes in Firearm Death Rates from 1989 to 1993 vs 2015 to 2019

		Mean (SD) [Range][a]		
		Early period, 5y (1989-1993)	Recent period, 5y (2015-2019)	Mean Change for 2015-2019 vs 1989-1993[a]
Firearm death (Rate, No./100 000 population)[b]		13.97 (8.31) [0-85.04]	14.13 (8.2) [0-115.61]	0.16 (8.78) [-76.58-102.79]
	0-19y	5.06 (7.37) [0-123.76]	3.92 (6.15) [0-89.29]	-1.14 (9.12) [-123.76-75.64]
	20-34y	20.55 (19.36) [0-308.17]	20.14 (24.25) [0-625]	-0.41 (28.18) [-308.17-625]
	35-54y	15.92 (13.67) [0-185.53]	18.48 (15.79) [0-173.16]	2.57 (18.38) [-185.53-173.16]
	55y+	17.31 (12.95) [0-125.43]	16.59 (10.9) [0-106.04]	-0.72 (15.09) [-124.69-106.04]
Firearm homicide (Rate, No./100 000 population)[b]		3.53 (4.35) [0-56.45]	3.14 (4.39) [0-66.69]	-0.39 (3.96) [-42-43.79]
	0-19y	1.4 (3.67) [0-90.12]	1.22 (3.02) [0-42.04]	-0.19 (4.11) [-90.12-42.04]
	20-34y	7.2 (11.25) [0-154.08]	6.6 (12.91) [0-362.98]	-0.6 (13.29) [-154.08-362.98]
	35-54y	4.64 (6.63) [0-74.82]	4.23 (6.78) [0-86.58]	-0.41 (7.82) [-74.82-86.58]
	55y+	1.85 (3.76) [0-55.34]	1.38 (2.75) [0-47.51]	-0.47 (4.16) [-55.34-41.1]
Firearm suicide (Rate, No./100 000 population)[b]		9.32 (5.87) [0-85.04]	10.53 (6.53) [0-68.95]	1.21 (6.91) [-64.26-68.95]
	0-19y	2.55 (5.22) [0-83.61]	2.36 (5) [0-89.29]	-0.18 (7.03) [-83.61-75.64]
	20-34y	11.59 (13.28) [0-238.38]	12.84 (19.58) [0-625]	1.25 (22.64) [-238.38-625]
	35-54y	10.23 (10.37) [0-185.53]	13.73 (13.33) [0-157.23]	3.51 (15.34) [-185.53-127.55]
	55y+	14.64 (11.66) [0-125.43]	14.82 (10.17) [0-106.04]	0.18 (14.01) [-124.69-106.04]

Abbreviations: SD, standard deviation; y, years; vs, versus.
[a] Figures are based on 3111 US counties.
[b] Rates for all ages represent age-standardized rates (per 100,000) while rates for age groups represent age-specific rates (per 100,000).

© 2022 Degli Esposti MD et al. *JAMA Network Open.*

eTable 5. Largest Decreases and Increases in County-Level Firearm Homicide Rates, 1989 to 1993 vs 2015 to 2019

Rank	Largest decreases in firearm homicide rates (per 100 000)						Largest increases in firearm homicide rates (per 100 000)					
	County name	State	Population size[a]	Age-standardized rate, 1989-1993	Age-standardized rate, 2015-2019	Rate change (Percentage change (%)[b])	County name	State	Population size[a]	Age-standardized rate, 1989-1993	Age-standardized rate, 2015-2019	Rate change (Percentage change (%)[b])
1	District of Columbia	DC	550521	56.45	14.45	-42 (-74.41)	Terrell County	TX	996	*	*	43.79 (..)
2	Daggett County	UT	943	*	*	-28.72 (..)	Petersburg City	VA	32604	13.94	41.39	27.45 (197)
3	Edwards County	TX	1987	*	*	-21.71 (..)	Allendale County	SC	10917	*	*	22.2 (246.73)
4	Cumberland County	VA	9378	*	*	-21.42 (-91.12)	St. Louis City	MO	344362	45.06	66.69	21.63 (47.99)
5	Storey County	NV	4074	*	*	-21.23 (-88.66)	Montgomery County	MS	11829	*	*	21.25 (1127.73)
6	Oldham County	TX	2118	*	*	-20.92 (..)	Dallas County	AL	44366	6.32	27.34	21.03 (332.78)
7	Richmond City	VA	193777	48.59	28.17	-20.42 (-42.03)	Hampton County	SC	21329	*	23.78	20.49 (623.12)
8	Bronx County	NY	1357589	23.59	3.46	-20.14 (-85.34)	Rich County	UT	2051	*	*	20.13 (..)
9	Boise County	ID	7535	*	*	-18.4 (..)	Baltimore City	MD	635815	29.71	47.43	17.72 (59.64)
10	Jackson County	TN	11072	*	*	-18.13 (..)	Irion County	TX	1756	*	*	17.54 (..)
11	New York County	NY	1593200	18.46	1.33	-17.13 (-92.79)	Graham County	KS	2721	*	*	17.32 (..)
12	Kings County	NY	2486235	19.81	2.97	-16.84 (-85.01)	Carter County	MT	1320	*	*	17 (185.97)
13	Houston County	TN	7988	*	*	-16.78 (..)	Lincoln County	MS	33906	*	*	15.69 (791.27)
14	Prince Edward County	VA	20455	*	*	-15.87 (-87.67)	Macon County	AL	22810	16.97	32.64	15.67 (92.29)
15	Leslie County	KY	11994	*	*	-15.78 (-68.98)	Bamberg County	SC	15880	*	*	15.17 (499.39)
16	Culberson County	TX	2627	*	*	-15.15 (..)	Madison Parish	LA	12457	*	*	15.1 (189.85)
17	Motley County	TX	1299	*	*	-14.46 (..)	Adams County	MS	32099	7.43	22.08	14.65 (197.14)
18	Hot Springs County	WY	4537	*	*	-13.98 (..)	Taliaferro County	GA	1826	*	*	14.42 (..)
19	Hyde County	NC	5413	*	*	-13.77 (..)	Comanche County	KS	1935	*	*	14.28 (..)
20	Mason County	TX	3880	*	*	-13.18 (..)	Wilkinson County	MS	10269	*	*	14.11 (113.78)
21	Edmunds County	SD	4112	*	*	-12.96 (..)	Foard County	TX	1518	*	*	14.05 (..)
22	Crenshaw County	AL	13727	*	*	-12.94 (-69.65)	Huerfano County	CO	7771	*	*	13.74 (581.02)
23	Kimble County	TX	4591	*	*	-12.92 (..)	Tunica County	MS	10321	*	*	13.54 (145.59)
24	Orleans Parish	LA	454863	52.13	39.43	-12.7 (-24.36)	Danville City	VA	46143	9.05	22.58	13.53 (149.53)
25	Howard County	AR	14552	*	*	-12.5 (..)	Grenada County	MS	22861	9.67	22.56	12.89 (133.31)

[a] Population sizes were based on estimates from 2005 (i.e., study period mid-point).

[b] Percentage change (%) is not available for counties that have rates that equal zero. For example, dividing by zero produces unmeaningful estimates (i.e., infinity).*Rates based on small counts (n<10), or which could be derived from rate change, were suppressed to preserve data confidentiality.

© 2022 Degli Esposti MD et al. *JAMA Network Open.*

eTable 6. Largest Decreases and Increases in County-Level Firearm Suicide Rates, 1989 to 1993 vs 2015 to 2019

Rank	Largest decreases in firearm suicide rates (per 100 000)						Largest increases in firearm suicide rates (per 100 000)					
	County name	State	Population size[a]	Age-standardized rate, 1989-1993	Age-standardized rate, 2015-2019	Rate change (Percentage change (%)[b])	County name	State	Population size[a]	Age-standardized rate, 1989-1993	Age-standardized rate, 2015-2019	Rate change (Percentage change (%)[b])
1	King County	TX	307	*	*	-64.26 (..)	Keya Paha County	NE	902	*	*	68.95 (..)
2	Meagher County	MT	1999	*	*	-55.04 (-76.01)	McMullen County	TX	883	*	*	55.85 (..)
3	Alpine County	CA	1159	*	*	-49.63 (..)	Terrell County	TX	996	*	*	40.56 (316.56)
4	Hinsdale County	CO	765	*	*	-46.88 (..)	Sherman County	OR	1749	*	*	37.8 (189.23)
5	Keweenaw County	MI	2195	*	*	-33.02 (..)	Harney County	OR	6898	*	*	37.29 (1220.24)
6	Wheeler County	OR	1455	*	*	-32.97 (-38.77)	Blaine County	NE	484	*	*	36.75 (..)
7	Throckmorton County	TX	1618	*	*	-32.47 (-74.65)	Granite County	MT	2965	*	*	35.52 (137.39)
8	Sanborn County	SD	2541	*	*	-29.46 (..)	Haakon County	SD	1912	*	*	35.29 (342.56)
9	Sheridan County	ND	1430	*	*	-28.91 (..)	Esmeralda County	NV	787	*	*	34.25 (236.76)
10	Garfield County	NE	1816	*	*	-28.27 (..)	Custer County	CO	3860	*	*	34.01 (297.4)
11	Ziebach County	SD	2631	*	*	-24.95 (..)	La Paz County	AZ	20238	*	*	31.99 (..)
12	Winchester City	VA	25119	39.96	15.22	-24.74 (-61.9)	Harding County	SD	1218	*	*	31.28 (..)
13	Pershing County	NV	6360	*	*	-24.38 (-59.95)	Deer Lodge County	MT	8948	*	*	31.22 (370.62)
14	Cottle County	TX	1746	*	*	-24.28 (..)	Adams County	ID	3591	*	*	30.57 (230.22)
15	Lafayette County	FL	7953	*	*	-23.2 (-92.47)	Loving County	TX	62	*	*	29.3 (..)
16	Hall County	TX	3700	*	*	-23.01 (-63.46)	Sierra County	CA	3434	*	*	27.74 (124.82)
17	Furnas County	NE	5019	*	*	-22.4 (-86.12)	McPherson County	NE	507	*	*	27.28 (..)
18	Golden Valley County	MT	1159	*	*	-21.29 (..)	Edwards County	TX	1987	*	*	26.9 (227.69)
19	Harlan County	NE	3462	*	*	-21.25 (-80.97)	Lane County	KS	1894	*	*	26.82 (..)
20	Fulton County	KY	7217	*	*	-20.88 (-76.36)	Piute County	UT	1365	*	*	26.59 (..)
21	Oldham County	TX	2118	*	*	-19.51 (..)	Petroleum County	MT	470	*	*	26.34 (..)
22	Webster County	NE	3762	*	*	-19.41 (-67.12)	Carter County	MT	1320	*	*	26.13 (..)
23	Storey County	NV	4074	*	*	-19.22 (-38.53)	Boise County	ID	7535	*	*	24.46 (430.83)
24	Fall River County	SD	7355	*	*	-18.23 (-67.05)	Kimble County	TX	4591	*	*	24.21 (202.96)
25	Covington City	VA	6205	*	*	-17.74 (-71.58)	Kittson County	MN	4792	*	*	23.91 (..)

[a] Population sizes were based on estimates from 2005 (i.e., study period mid-point).
[b] Percentage change (%) is not available for counties that have rates that equal zero. For example, dividing by zero produces unmeaningful estimates (i.e., infinity).
*Rates based on small counts (n<10), or which could be derived from rate change, were suppressed to preserve data confidentiality.

© 2022 Degli Esposti MD et al. *JAMA Network Open.*

eTable 7. Low and High County Outliers for Firearm Homicide Rates, 2015 to 2019

	County name	State	Age-standarized rate, 2015-2019		State firearm deaths (%)[c]	County characteristics[a]														
										Population demographics (%)										
			Observed	Expected (95% CrI)		Population size	RUCC	Land area (square miles)	Sex ratio (M/F)	Aged 15-65y	Black	Hispanic	High school graduate	Median household income ($)	Unemployment rate[d] (%)	Poverty (%)	Republican voters (%)	Heavy drinkers (%)	Trauma care access[e]	Firearm licences[f]
**Low outliers (below-expected rates in 2015-2019)**																				
	District of Columbia	DC	14.45	18.8 (15.54-22.12)	100	550521	1	61	0.9	70	57	9	87	48078	7	18	9	12	6	6
	Crenshaw County	AL	*	8.89 (5.8-12.01)	0.17	13727	8	610	0.91	64	25	1	74	29410	4	24	69	15	7	0
	Franklin County	MS	*	6.99 (3.83-10.2)	0.07	8411	9	565	0.93	66	36	1	78	29519	9	22	64	15	6	0
	Amite County	MS	*	6.06 (2.99-9.18)	0.07	13435	8	730	0.95	66	42	2	74	28549	9	21	58	15	10	0
	Cumberland County	VA	*	5.3 (2.18-8.46)	0.06	9378	1	298	0.93	66	34	2	73	34447	4	16	58	11	8	2
	Bullock County	AL	*	5.08 (1.96-8.24)	0.04	11055	6	625	1.15	68	71	6	75	21728	8	38	32	16	5	0
	Boise County	ID	*	3.21 (0.06-6.4)	0	7535	2	1902	1.07	71	0	4	87	42459	5	12	71	16	11	0
	Tensas Parish	LA	*	5.3 (2.19-8.45)	0	6125	9	602	1	66	57	2	71	23441	13	37	49	14	1	0
	Houston County	TN	*	3.61 (0.46-6.81)	0	7988	8	200	0.99	64	3	2	80	32590	9	18	40	14	1	1
	Jackson County	TN	*	3.36 (0.22-6.56)	0	11072	8	309	0.97	68	1	1	69	29966	9	21	40	14	4	0
	Culberson County	TX	*	3.34 (0.19-6.54)	0	2627	9	3812	0.97	63	1	71	62	26228	4	26	52	15	1	0
	Edwards County	TX	*	3.93 (0.83-7.09)	0	1987	9	2120	1.05	67	3	46	68	27942	4	29	77	16	1	0
	Oldham County	TX	*	4.34 (1.2-7.54)	0	2118	8	1501	1.07	64	3	13	82	36521	4	14	87	11	1	0

© 2022 Degli Esposti MD et al. *JAMA Network Open.*

	Daggett County	UT	*	4.97 (1.75-8.26)	0	943	8	698	1.3	66	1	6	85	36021	5	7	76	17	2	0
**High outliers (above-expected rates in 2015-2019)**																				
	Baltimore City	MD	47.43	39.7 (35.83-43.41)	66.73	635815	1	81	0.87	67	65	2	77	32453	7	22	17	13	..	7
	Jefferson County	KY	13	9.4 (6.32-12.44)	45.23	699827	1	385	0.93	67	20	2	87	40973	6	14	49	11	104	1
	Marion County	IN	16.35	11.89 (8.73-14.99)	43.95	863133	1	396	0.94	66	26	6	84	42129	5	15	49	11	129	2
	Shelby County	TN	22.52	18.86 (15.71-21.97)	42.85	909035	1	755	0.92	68	51	3	85	40917	6	19	42	15	159	2
	St. Louis City	MO	66.69	54.12 (49.85-58.19)	39.17	344362	1	62	0.9	67	51	2	81	30629	8	26	19	15	..	3
	Jefferson County	AL	22.72	18.9 (15.75-22.01)	31.35	657229	1	1113	0.9	67	41	2	87	41821	4	16	54	14	138	1
	Orleans Parish	LA	39.43	35.29 (31.96-38.54)	30.18	454863	1	181	0.89	67	68	3	83	30216	..	26	22	12	31	1
	Wyandotte County	KS	20.09	16.84 (13.65-19.99)	28.83	155750	1	151	0.96	66	27	21	79	34593	9	20	34	13	29	4
	Jackson County	MO	20.6	16.14 (12.98-19.25)	25.82	662959	1	605	0.94	67	23	7	87	43284	6	14	41	11	176	4
	Hinds County	MS	32.7	26.54 (23.29-29.73)	25.37	249345	2	869	0.9	67	65	1	84	35433	7	22	40	14	85	0
	East Baton Rouge Parish	LA	21.39	18.05 (14.9-21.15)	18.04	411417	2	455	0.93	69	43	2	87	39661	7	21	54	11	80	0
	Montgomery County	AL	22.57	18.12 (14.95-21.23)	10.57	221619	2	790	0.92	67	52	1	85	39749	4	17	49	15	57	0
	Jefferson County	AR	26.61	20.58 (17.29-23.79)	9.83	81700	3	885	0.96	67	52	1	82	32105	7	21	34	13	41	1

© 2022 Degli Esposti MD et al. *JAMA Network Open.*

	Newport News City	VA	16.56	12.16 (9.02-15.26)	8.86	179899	1	68	0.92	65	42	5	89	43938	4	14	47	11	33	2
	Charles Mix County	SD	*	7.2 (4.11-10.25)	8.47	9194	9	1098	0.97	59	0	2	81	29778	4	30	53	15	9	0
	Bottineau County	ND	*	8.48 (5.2-11.69)	5.17	6741	9	1669	1.03	66	0	1	86	35999	4	12	67	13	14	0
	Mississippi County	AR	21.15	16.28 (13.17-19.35)	4.92	47911	4	898	0.92	64	34	3	76	29822	8	24	43	14	14	1
	Muscogee County	GA	16.17	12.75 (9.56-15.89)	4.82	185271	2	216	0.93	66	46	4	84	34954	6	20	48	13	48	0
	Washington County	MS	32.24	27.65 (24.44-30.81)	4.74	59220	5	724	0.87	64	67	1	73	25155	12	36	39	12	34	1
	Robeson County	NC	18.94	15.81 (12.74-18.84)	4.57	127586	4	949	0.97	66	24	7	69	27159	7	32	47	13	24	0
	Bibb County	GA	18.37	15.07 (11.89-18.21)	4.09	154918	3	250	0.85	65	50	2	81	34872	6	24	49	12	40	1
	Petersburg City	VA	41.39	30.76 (26.89-34.47)	3.94	32604	1	23	0.84	62	79	2	72	30942	7	22	19	11	..	1
	Deer Lodge County	MT	*	7.85 (4.65-10.99)	3.81	8948	7	737	1.01	68	0	2	85	30579	6	16	38	18	14	0
	Phillips County	AR	32.31	26.2 (22.93-29.4)	3.24	24107	7	693	0.85	60	61	2	71	24141	9	34	36	14	11	2
	Danville City	VA	22.58	18.61 (15.1-22.03)	2.79	46143	3	43	0.84	61	46	2	76	29544	10	24	49	9	..	3
	Leflore County	MS	30.08	25.55 (22.33-28.7)	2.74	36431	5	592	0.92	65	71	2	69	22640	10	37	37	14	14	1
	Adams County	MS	22.08	17.99 (14.75-21.16)	2.27	32099	5	460	0.86	64	56	1	80	27232	9	27	45	15	19	0
	Dallas County	AL	27.34	20.76 (17.4-24.03)	2.22	44366	4	981	0.84	64	67	1	77	24936	8	35	39	13	16	1

© 2022 Degli Esposti MD et al. *JAMA Network Open.*

	Coahoma County	MS	27.36	23.47 (20.29-26.6)	2.2	29002	5	554	0.86	62	73	1	74	23728	11	40	35	13	15	2
	Lincoln County	MS	17.67	13.3 (10.15-16.4)	1.94	33906	6	586	0.93	66	30	1	82	31441	8	21	69	13	15	0
	Sweet Grass County	MT	*	7.24 (4.02-10.4)	1.9	3672	9	1855	1.02	66	0	2	93	36981	2	11	76	12	9	0
	Eddy County	ND	*	8.26 (5.06-11.42)	1.72	2626	9	630	0.93	59	0	1	82	32576	6	11	54	12	3	0
	Mellette County	SD	*	7.5 (4.35-10.6)	1.69	2088	9	1306	1.02	61	0	2	86	28439	6	33	59	20	1	0
	Clay County	WV	*	12.77 (9.55-15.94)	1.67	10356	2	342	1	67	0	0	73	25945	8	27	54	13	11	1
	Grenada County	MS	22.56	18.81 (15.68-21.9)	1.54	22861	7	422	0.89	65	42	1	74	30076	8	22	58	13	13	1
	Vance County	NC	18.97	15.19 (11.92-18.4)	1.5	43771	4	254	0.9	64	50	6	73	32133	9	23	44	11	9	2
	Macon County	AL	32.64	25.23 (21.82-28.55)	1.26	22810	6	611	0.86	67	83	1	79	23500	5	32	17	15	8	0
	Hampton County	SC	23.78	18.69 (15.45-21.86)	1.15	21329	6	560	1.04	67	56	3	76	31309	8	24	39	14	9	1
	Carter County	MT	*	18.24 (14.81-21.58)	0.95	1320	9	3340	0.97	68	0	1	91	29496	4	13	88	13	2	0
	Rich County	UT	*	12.56 (9.22-15.81)	0.91	2051	8	1029	1.04	64	0	2	95	45335	3	10	89	15	3	2
	Tunica County	MS	22.83	18.41 (15.27-21.49)	0.73	10321	1	455	0.89	65	73	3	72	26370	11	28	30	13	4	2
	Montgomery County	MS	23.14	17.9 (14.58-21.13)	0.73	11829	7	407	0.89	63	46	1	75	28349	8	23	55	12	5	1
	Wilkinson County	MS	26.52	21.28 (18.03-24.47)	0.73	10269	8	677	1.12	67	70	0	69	21904	13	37	36	17	4	0

© 2022 Degli Esposti MD et al. *JAMA Network Open.*

	Allendale County	SC	31.2	24.25 (20.85-27.56)	0.71	10917	6	408	1.11	66	72	2	73	22491	11	38	27	13	3	2
	McDuffie County	GA	19.23	14.4 (11.18-17.56)	0.61	21743	2	260	0.91	66	38	2	70	33987	7	21	62	13	11	1
	Sumter County	GA	13.91	9.86 (6.76-12.91)	0.61	32912	6	485	0.9	64	50	3	75	30996	7	28	50	14	22	1
	Huerfano County	CO	*	11.89 (8.7-15.02)	0.49	7771	6	1591	1.16	66	3	35	85	28334	8	23	50	13	10	0
	Wilcox County	AL	19.72	16.4 (13.24-19.5)	0.48	12937	8	889	0.85	63	73	1	69	19407	8	38	32	15	5	0
	Texas County	MO	10.73	7.53 (4.47-10.56)	0.48	24614	9	1179	1.02	65	2	1	79	27893	7	22	66	15	29	0
	Madison Parish	LA	23.05	18.11 (14.84-21.31)	0.47	12457	7	624	0.98	65	61	3	73	22951	9	37	49	15	9	0
	St. Helena Parish	LA	18.81	15.59 (12.4-18.73)	0.39	10259	2	408	0.94	67	54	1	77	28952	13	22	41	13	2	1
	Stafford County	KS	*	8.48 (5.3-11.61)	0.36	4488	9	792	0.97	62	0	7	88	34077	4	14	75	11	2	0
	Graham County	KS	*	11.5 (8.21-14.71)	0.36	2721	9	898	0.98	60	3	1	92	33029	3	12	75	12	5	0
	Pulaski County	GA	*	14.96 (11.74-18.13)	0.27	9737	6	247	0.76	68	32	4	77	33333	6	20	63	15	5	1
	Atkinson County	GA	*	12.09 (8.84-15.27)	0.21	8030	9	338	0.98	64	18	22	67	27657	5	25	67	15	4	0
	Comanche County	KS	*	9.75 (6.5-12.93)	0.18	1935	9	788	0.91	60	0	2	93	31964	3	11	78	12	5	0
	Trinity County	CA	*	11.49 (8.26-14.66)	0.12	13622	8	3179	1.05	68	1	5	90	31434	10	16	55	20	15	0
	Lynn County	TX	*	7.29 (4.18-10.36)	0.05	6237	6	892	1	62	3	46	74	31207	6	21	78	10	2	1

© 2022 Degli Esposti MD et al. *JAMA Network Open.*

	Taliaferro County	GA	*	10.08 (6.84-13.26)	0.03	1826	8	195	0.91	64	61	1	58	24893	7	29	35	12	1	1
	Donley County	TX	*	6.98 (3.87-10.05)	0.03	3889	8	930	0.95	62	5	7	82	30561	5	19	80	11	2	0
	Terrell County	TX	*	30.51 (26.31-34.52)	0.03	996	9	2358	0.97	64	0	51	80	27927	7	20	65	10	1	0
	Foard County	TX	*	9.12 (5.9-12.27)	0.02	1518	9	707	0.92	58	4	18	76	25535	5	18	59	10	1	0
	Irion County	TX	*	12.49 (9.14-15.77)	0.02	1756	3	1051	0.97	68	2	27	82	40709	4	9	83	11	2	0

Abbreviations: CrI, credible intervals; F, female; M, male; RUCC, rural-urban continuum code; SD, standard deviation; y, years.
[a] Supplement eTable 1 for details on county characteristics. Timings of measures range from 1999-2010, with most characteristics measured in 2005.
[b] Expected age-standardised mortality rates were estimated from our Bayesian models for all counties (n=3111).
[c] The percentage of state firearm deaths that occur in rural counties in 2015-2019 accounted for by that county outlier.
[d] Number unemployed as a percent of the labor force, see Supplement eTable 1.
[e] Number of Level 1 Trauma Centers within 60 miles (crow-fly distance).
[f] Per capita prevalence of "type one" (firearm dealer) and "type two" (pawnbroker) federal firearm licenses.
*Rates based on small counts (n<10) were suppressed to preserve data confidentiality.

© 2022 Degli Esposti MD et al. *JAMA Network Open.*

eTable 8. Low and High County Outliers for Firearm Suicide Rates, 2015 to 2019

	County name	State	Age-standarized rate, 2015-2019		State firearm deaths (%)[c]	County characteristics[a]														
										Population demographics (%)										
			Observed	Expected (95% CrI)		Population size	RUCC	Land area (square miles)	Sex ratio (M/F)	Aged 15-65y	Black	Hispanic	High school graduate	Median household income ($)	Unemployment rate[d] (%)	Poverty (%)	Republican voters (%)	Heavy drinkers (%)	Trauma care access[e]	Firearm licences[f]
**Low outliers (below-expected rates in 2015-2019)**																				
	Furnas County	NE	*	8.75 (3.63-13.96)	0.15	5019	9	718	0.91	60	0	1	87	33011	4	12	79	11	6	0
	Meagher County	MT	*	23.26 (18.08-28.56)	0.11	1999	9	2392	1	64	0	2	82	29099	4	17	72	19	11	0
	Alpine County	CA	*	7.08 (1.81-12.5)	0	1159	8	739	1.11	74	1	9	92	45283	8	17	44	25	1	0
	Hinsdale County	CO	*	10.02 (4.58-15.64)	0	765	9	1118	1.01	68	0	2	91	42012	3	9	59	18	4	0
	Keweenaw County	MI	*	5.8 (0.57-11.15)	0	2195	9	541	1.1	64	2	1	91	31809	11	15	54	20	1	0
	Golden Valley County	MT	*	7.16 (1.91-12.55)	0	1159	8	1175	1.04	65	0	1	87	27455	4	20	76	18	1	0
	Sheridan County	ND	*	6.72 (1.5-12.08)	0	1430	9	972	1.03	61	0	0	80	29229	6	20	77	10	0	0
	Brown County	NE	*	5.35 (0.17-10.65)	0	3328	9	1221	0.97	60	0	1	88	32291	3	13	82	11	7	0
	Sanborn County	SD	*	5.19 (0.07-10.42)	0	2541	9	569	1.05	64	0	1	85	35269	3	14	57	15	1	0
	Ziebach County	SD	*	5.98 (0.8-11.27)	0	2631	9	1962	0.97	65	0	1	77	21213	8	51	40	29	1	0
	Cottle County	TX	*	5.24 (0.12-10.45)	0	1746	9	901	0.89	57	12	22	79	28011	6	21	71	11	3	0
	King County	TX	*	8.92 (3.56-14.46)	0	307	9	912	1.31	72	0	15	91	41738	4	14	88	13	2	0
**High outliers (above-expected rates in 2015-2019)**																				
	Morgan County	WV	27.89	22.04 (16.7-27.25)	2	16022	3	229	0.99	66	1	1	84	40171	5	11	66	11	8	1

© 2022 Degli Esposti MD et al. *JAMA Network Open.*

	Deer Lodge County	MT	39.64	33.33 (27.97-38.55)	1.99	8948	7	737	1.01	68	0	2	85	30579	6	16	38	18	14	0
	Kingsbury County	SD	*	23.56 (18.17-28.82)	1.85	5532	9	838	0.95	61	0	1	87	35604	4	11	60	11	11	0
	Ransom County	ND	*	21.5 (16.23-26.67)	1.67	5810	8	863	1.05	64	0	1	87	42714	3	10	52	12	12	0
	Stanley County	SD	*	22.88 (17.63-28.02)	1.15	2829	9	1443	0.98	69	0	0	91	46152	3	9	70	12	1	0
	Phillips County	MT	40.01	34.19 (28.84-39.41)	1.1	4179	9	5140	1.01	64	0	1	88	32309	4	18	77	14	13	0
	Haakon County	SD	*	34.2 (28.45-39.75)	0.92	1912	8	1813	0.99	63	0	1	88	35183	3	11	81	13	4	0
	Granite County	MT	*	50.08 (44.16-55.77)	0.88	2965	8	1727	1.03	68	0	1	91	32063	5	15	71	19	6	0
	Garfield County	UT	49.05	40.26 (34.72-45.63)	0.86	4470	9	5174	1.06	60	0	3	92	38751	7	10	85	17	7	0
	La Paz County	AZ	31.99	23.95 (18.51-29.23)	0.85	20238	6	4500	1.03	55	1	23	77	29015	7	22	62	20	2	0
	Adams County	ID	*	34.97 (29.43-40.33)	0.79	3591	8	1365	1.03	68	0	2	88	38028	8	14	71	16	12	0
	Musselshell County	MT	*	36.69 (31.26-41.97)	0.77	4497	8	1867	0.94	67	0	2	86	30386	5	18	74	15	10	0
	Catron County	NM	44.67	37.25 (31.83-42.52)	0.75	3409	9	6928	1	64	0	19	86	25917	7	23	72	15	14	0
	Harney County	OR	40.35	30.9 (25.35-36.28)	0.7	6898	7	10134	1.05	64	0	4	90	33795	9	15	76	16	14	0
	Buffalo County	SD	*	24.08 (18.73-29.31)	0.69	2100	9	471	0.99	61	0	1	77	16868	15	39	27	33	0	0

© 2022 Degli Esposti MD et al. *JAMA Network Open.*

	Sweet Grass County	MT	*	27.67 (22.34-32.89)	0.66	3672	9	1855	1.02	66	0	2	93	36981	2	11	76	12	9	0
	Clear Creek County	CO	40.32	33.99 (28.61-39.24)	0.63	9197	1	395	1.07	75	0	4	97	61937	5	7	45	16	8	2
	Gosper County	NE	*	26.89 (21.43-32.2)	0.61	2020	9	458	1.02	61	0	1	94	41688	4	8	80	10	4	0
	Stafford County	KS	*	26.36 (21.02-31.57)	0.51	4488	9	792	0.97	62	0	7	88	34077	4	14	75	11	2	0
	Tillman County	OK	29.18	23.41 (18.12-28.58)	0.47	8513	6	872	0.95	62	8	19	74	27228	5	24	66	10	3	0
	Keya Paha County	NE	*	47.8 (40.86-54.48)	0.46	902	9	773	1	58	0	5	91	31082	3	19	81	10	2	0
	Harding County	SD	*	23.77 (18.36-29.04)	0.46	1218	9	2671	1.05	68	0	1	90	31327	3	14	86	11	1	0
	Trego County	KS	*	38.54 (32.92-43.97)	0.44	3050	9	888	0.91	60	0	1	89	31258	3	13	73	12	1	0
	Republic County	KS	*	20.78 (15.48-25.96)	0.44	5164	9	716	0.96	59	0	1	95	31364	4	10	77	12	8	0
	Daviess County	MO	29.85	23.78 (18.46-28.96)	0.35	8121	8	567	0.93	63	0	1	84	33940	5	17	62	13	11	4
	Custer County	CO	45.45	37.07 (31.46-42.51)	0.33	3860	8	739	1.03	67	0	3	94	40946	5	13	68	13	4	0
	Huerfano County	CO	33.79	28.6 (23.34-33.75)	0.33	7771	6	1591	1.16	66	3	35	85	28334	8	23	50	13	10	0
	Hamilton County	KS	*	25.08 (19.74-30.29)	0.29	2604	9	996	0.98	62	1	27	81	34324	3	13	79	12	3	1
	Graham County	KS	*	24.87 (19.58-30.03)	0.29	2721	9	898	0.98	60	3	1	92	33029	3	12	75	12	5	0

© 2022 Degli Esposti MD et al. *JAMA Network Open.*

	Clay County	TN	26	20.62 (15.39-25.74)	0.28	7992	8	236	0.97	68	2	2	72	25865	12	22	49	14	3	0
	Rich County	UT	*	27.55 (22.33-32.67)	0.25	2051	8	1029	1.04	64	0	2	95	45335	3	10	89	15	3	2
	Sherman County	OR	*	45.91 (39.83-51.72)	0.23	1749	9	823	1	64	0	7	90	38806	7	16	63	17	6	0
	Clark County	ID	*	39.83 (34.43-45.09)	0.17	943	8	1765	1.13	62	0	39	69	32687	5	20	86	14	2	0
	Lane County	KS	*	21.48 (16.12-26.72)	0.15	1894	9	717	1	62	0	2	88	35857	4	9	81	11	3	0
	Holt County	MO	*	18.68 (13.42-23.84)	0.15	5081	8	462	1	62	0	0	89	32146	5	13	69	12	6	0
	Blaine County	NE	*	27.06 (21.46-32.49)	0.15	484	9	711	1.08	61	0	0	96	31533	4	18	89	9	0	0
	McPherson County	NE	*	20.66 (15.31-25.88)	0.15	507	9	859	0.99	62	0	2	87	33677	2	14	83	11	0	0
	Wheeler County	OR	*	46.62 (41.28-51.85)	0.14	1455	9	1715	1.01	62	0	6	88	31525	6	18	70	16	3	0
	Esmeralda County	NV	*	37.96 (32.12-43.58)	0.12	787	9	3589	1.16	65	0	12	84	38527	5	15	76	25	3	0
	Carter County	MT	*	20.98 (15.73-26.12)	0.11	1320	9	3340	0.97	68	0	1	91	29496	4	13	88	13	2	0
	Cheyenne County	CO	*	29.26 (23.81-34.56)	0.1	1953	9	1781	0.97	64	1	9	88	39252	3	13	81	12	2	0
	Sierra County	CA	*	38.91 (33.1-44.5)	0.09	3434	8	953	1.02	67	0	8	88	39380	8	11	64	21	2	0
	Highland County	VA	*	24.8 (19.41-30.06)	0.09	2475	9	416	0.98	64	0	0	74	34519	3	13	65	19	7	0

© 2022 Degli Esposti MD et al. *JAMA Network Open.*

	San Juan County	CO	*	33.55 (28.28-38.73)	0.07	577	9	387	1.05	78	0	9	93	33462	8	17	44	19	2	0
	Mineral County	CO	*	51.6 (45.82-57.17)	0.07	932	9	876	1.05	66	0	2	97	40134	5	10	62	14	1	0
	Haskell County	TX	*	19.22 (13.99-24.34)	0.07	5541	6	903	0.88	59	4	24	78	26636	4	24	64	13	6	0
	Kimble County	TX	*	29.33 (24-34.54)	0.07	4591	7	1251	0.98	62	0	23	77	31185	4	19	82	13	4	0
	Menard County	TX	*	33.81 (28.36-39.11)	0.04	2201	8	902	1.01	61	1	32	80	27013	5	26	69	15	5	0
	Edwards County	TX	*	30.92 (25.51-36.19)	0.03	1987	9	2120	1.05	67	3	46	68	27942	4	29	77	16	1	0
	McMullen County	TX	*	40.49 (34.18-46.54)	0.02	883	8	1113	1.05	69	1	35	79	36046	5	15	83	15	1	2
	Terrell County	TX	*	41.72 (35.77-47.43)	0.02	996	9	2358	0.97	64	0	51	80	27927	7	20	65	10	1	0
	Borden County	TX	*	18.3 (13.07-23.43)	0.01	648	9	899	0.95	72	0	15	85	36817	4	9	84	16	2	1
	Loving County	TX	*	23.37 (18.02-28.6)	0.01	62	9	673	1	61	0	18	92	40785	10	25	81	15	0	0

Abbreviations: CrI, credible intervals; F, female; M, male; RUCC, rural-urban continuum code; SD, standard deviation; y, years.
[a] Supplement eTable 1 for details on county characteristics. Timings of measures range from 1999-2010, with most characteristics measured in 2005.
[b] Expected age-standardised mortality rates were estimated from our Bayesian models for all counties (n=3111).
[c] The percentage of state firearm deaths that occur in rural counties in 2015-2019 accounted for by that county outlier.
[d] Number unemployed as a percent of the labor force, see Supplement eTable 1.
[e] Number of Level 1 Trauma Centers within 60 miles (crow-fly distance).
[f] Per capita prevalence of "type one" (firearm dealer) and "type two" (pawnbroker) federal firearm licenses.
*Rates based on small counts (n<10) were suppressed to preserve data confidentiality.

© 2022 Degli Esposti MD et al. *JAMA Network Open.*

eTable 9. Posthoc Pairwise Comparisons of County Characteristics by Outlier Status (no outlier, low outlier, high outlier)

	Firearm death			Firearm homicide			Firearm suicide		
	**No vs low outlier**	**No vs high outlier**	**Low vs high outlier**	**No vs low outlier**	**No vs high outlier**	**Low vs high outlier**	**No vs low outlier**	**No vs high outlier**	**Low vs high outlier**
**Counties, N**	3029	15	67	3034	14	63	3046	12	53
**County Characteristic[a], *P* value[b]**									
**Geography**									
RUCC	<0.001	<0.001	0.016	..	..	..	<0.001	<0.001	0.068
Land area (square miles)	0.053	<0.001	0.9	..	..	..	0.010	<0.001	>0.9
**Sociodemographic**									
Sex ratio (M/F)	0.008	0.047	0.047	0.7	<0.001	0.014	0.079	<0.001	0.6
Aged 15-65y (%)	0.3	<0.001	0.6	0.7	0.012	0.2	0.11	<0.001	0.9
Black (%)	<0.001	<0.001	0.15	0.031	<0.001	0.3	<0.001	<0.001	0.2
Hispanic (%)	..	..	..	..	..	..	..	..	..
**Education**									
High school graduate (%)	..	..	..	<0.001	<0.001	0.13	0.3	0.002	0.6
**Economic**									
Median household income ($)	0.016	<0.001	>0.9	0.003	<0.001	>0.9	.031	<0.001	0.5
Unemployment rate[d] (%)	..	..	..	0.3	<0.001	0.7	..	..	..
Poverty (%)	..	..	..	0.003	<0.001	0.6	..	..	..
**Politics**									
Republican voters (%)	0.036	0.066	0.2	0.5	<0.001	0.3	0.14	<0.001	0.4
**Health**									
Heavy drinkers (%)	..	..	..	..	..	..	0.12	0.12	0.3
Trauma care access[e]	0.002	<0.001	0.15	..	..	..	<0.001	<0.001	0.2
**Firearms**									
Firearm licences[f]	<0.001	<0.001	<0.001	<0.001	0.034	0.003	<0.001	<0.001	0.12

Abbreviations: F, female; M, male; RUCC, rural-urban continuum code; SD, standard deviation; y, years.

[a] Supplement eTable 1 for details on county characteristics. Timings of measures range from 1999-2010, with most characteristics measured in 2005.

[b] Posthoc pairwise comparisons using Wilcoxon rank sum tests (Mann Whitney U). All *p* values adjusted for multiple testing by using the Benjamini-Hochberg method to control for the false discovery rate. See Table 3 in manuscript for mean differences.

[d] Number unemployed as a percent of the labor force, see Supplement eTable 1.

[e] Number of Level 1 Trauma Centers within 60 miles (crow-fly distance).

[f] Per capita prevalence of "type one" (firearm dealer) and "type two" (pawnbroker) federal firearm licenses.

© 2022 Degli Esposti MD et al. *JAMA Network Open.*

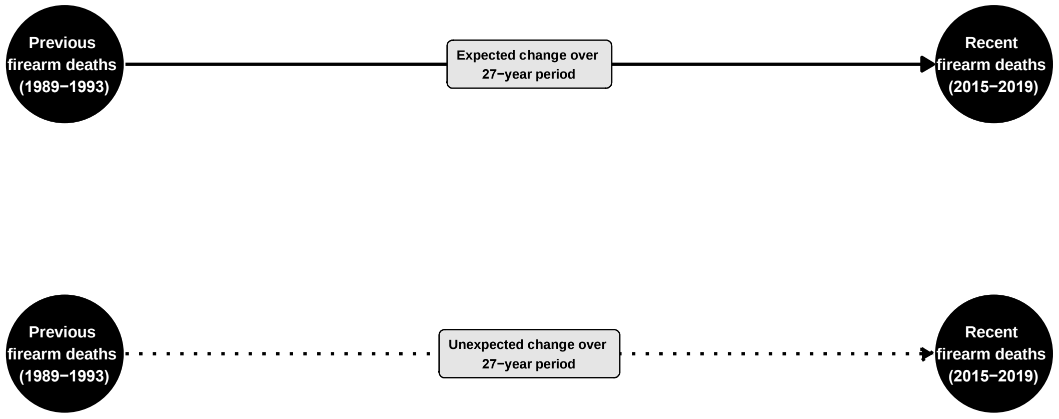

eFigure 1. Schematic Diagram Illustrating how the Relationship Between Previous and Recent Firearm Deaths can Capture Expected *(Top)* and Unexpected *(Bottom)* Change Over Time.

© 2022 Degli Esposti MD et al. *JAMA Network Open.*

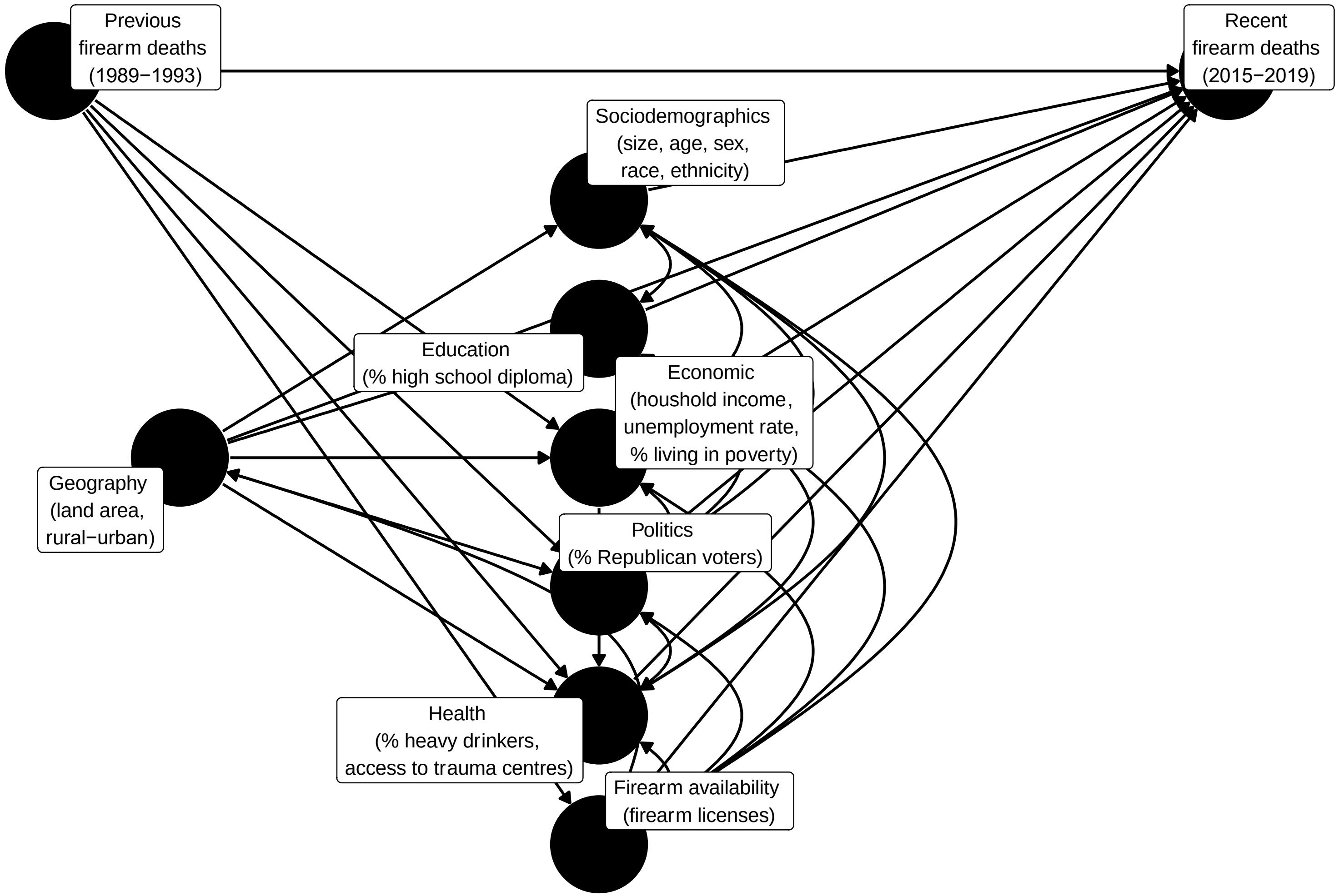

eFigure 2. Schematic Directed Acyclical Graph (DAG) of the Associations Between County-Level Covariates and Previous (1989 to 1993) and Recent (2015 to 2019) Firearm Deaths.

© 2022 Degli Esposti MD et al. *JAMA Network Open.*

Age−standardized firearm homicide rates, 1989−1993

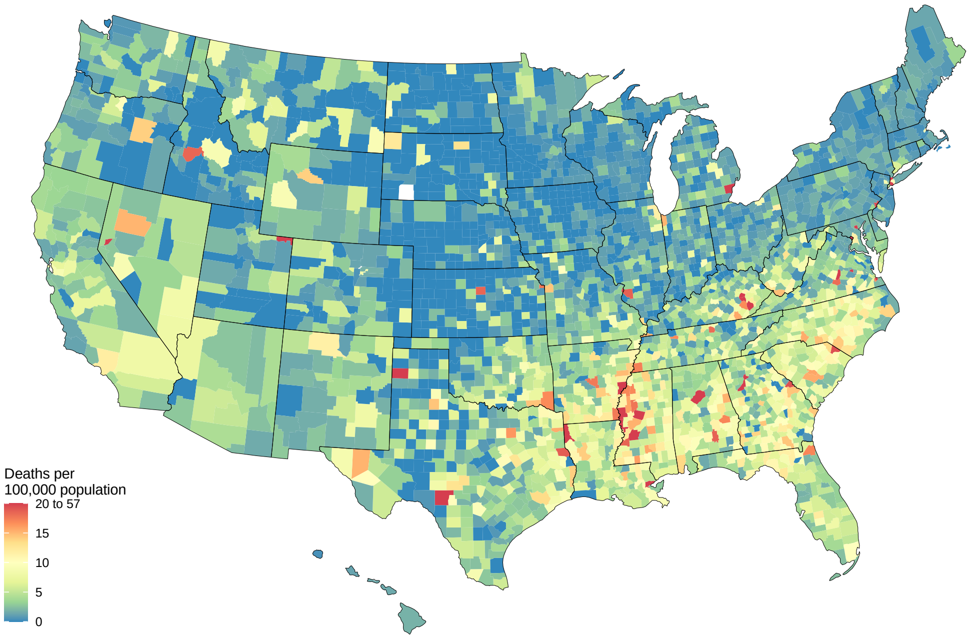

Age−standardized firearm homicide rates, 2015−2019

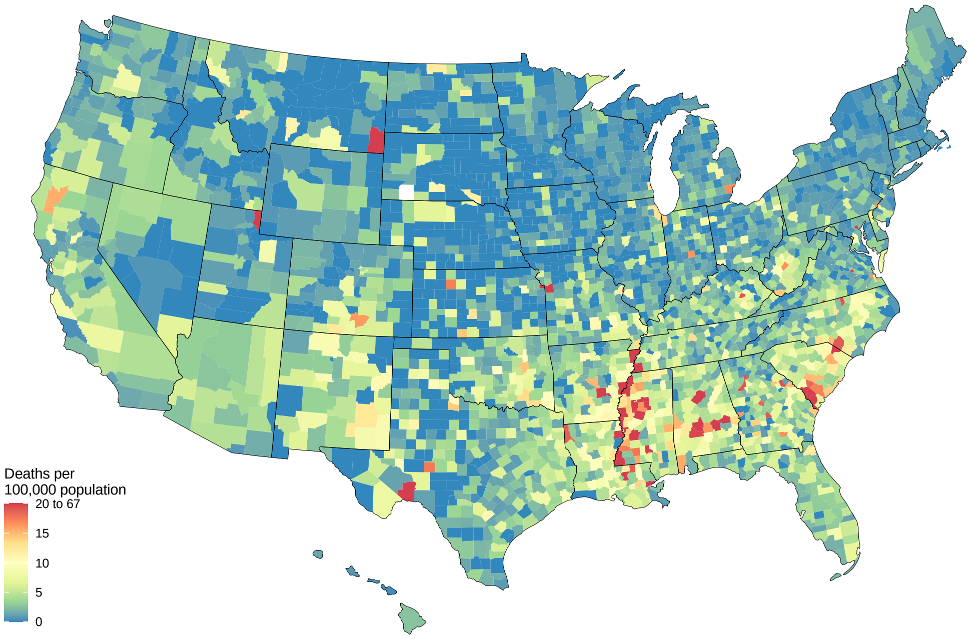

eFigure 3. Age-Standardized Firearm Homicide Rates in 1989 to 1993 and 2015 to 2019.

© 2022 Degli Esposti MD et al. *JAMA Network Open.*

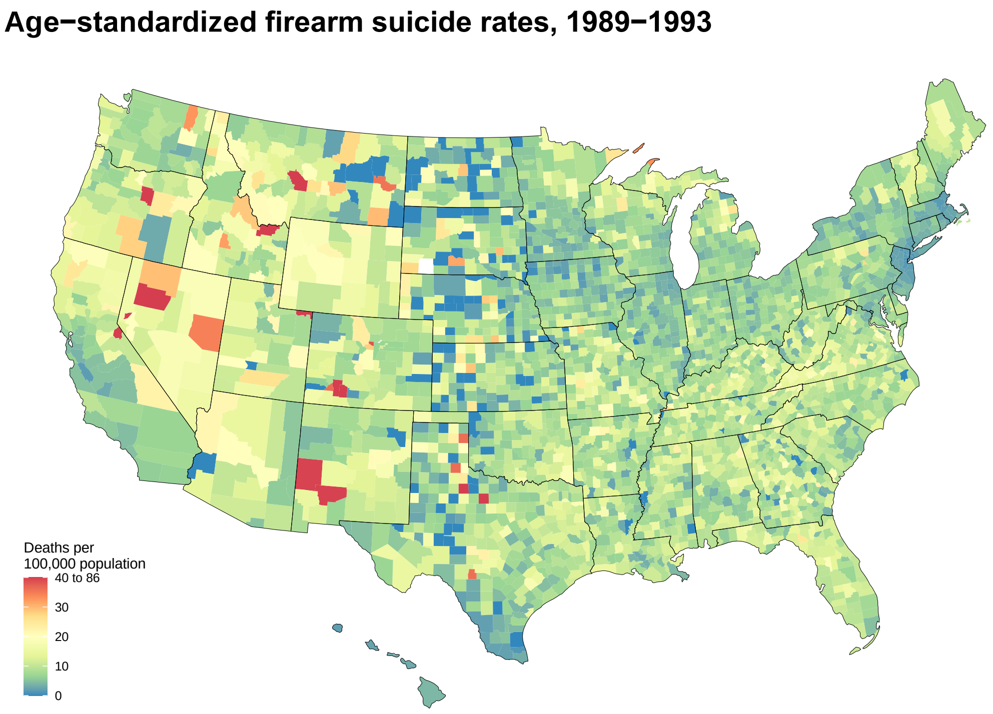

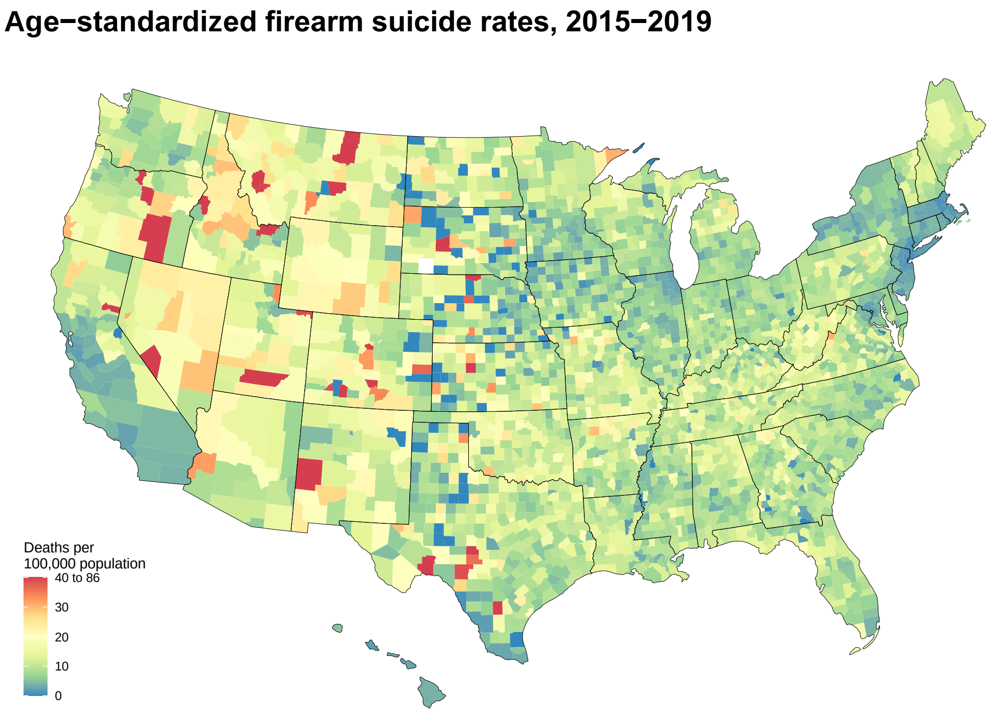

eFigure 4. Age-Standardized Firearm Suicide Rates in 1989 to 1993 and 2015 to 2019.

© 2022 Degli Esposti MD et al. *JAMA Network Open.*

eReferences

1. Besag J, York J, Mollié A. Bayesian image restoration, with two applications in spatial statistics. *Ann Inst Stat Math*. 1991;43(1):1-20.

2. Blangiardo M, Cameletti M, Baio G. A tutorial in spatial and spatio-temporal models with R-INLA. :38.

3. Rue H, Riebler A, Sørbye SH, Illian JB, Simpson DP, Lindgren FK. Bayesian Computing with INLA: A Review. *Annu Rev Stat Its Appl*. 2017;4(1):395-421. doi:10.1146/annurev-statistics-060116-054045

4. Rue H, Martino S, Chopin N. Approximate Bayesian inference for latent Gaussian models by using integrated nested Laplace approximations. *J R Stat Soc Ser B Stat Methodol*. 2009;71(2):319-392.

© 2022 Degli Esposti MD et al. *JAMA Network Open.*
